# The Transcription Co-Repressors MTG8 and MTG16 Regulate Exit of Intestinal Stem Cells From Their Niche and Differentiation Into Enterocyte vs Secretory Lineages

**DOI:** 10.1053/j.gastro.2020.06.012

**Published:** 2020-10

**Authors:** Anna Baulies, Nikolaos Angelis, Valentina Foglizzo, E. Thomas Danielsen, Harshil Patel, Laura Novellasdemunt, Anna Kucharska, Joana Carvalho, Emma Nye, Paolo De Coppi, Vivian S.W. Li

**Affiliations:** 1 The Francis Crick Institute, London, UK; 2 Department of Paediatric Surgery, UCL Great Ormond Street Institute of Child Health and Great Ormond Street Hospital for Children, London, UK

**Keywords:** Niche Exit, Lineage Specification, Chromatin Remodeling, Lateral Inhibition, ChIP-seq, chromatin immunoprecipitation coupled-deep sequencing, DBZ, dibenzazepine, DHS, DNase I hypersensitivity, DKO, double knockout, Dll, Delta like, GFP, green fluorescent protein, ISC, intestinal stem cell, MTG, myeloid translocating gene, qRT-PCR, quantitative reverse-transcriptase polymerase chain reaction, RNA-seq, RNA sequencing, RSPO, R-spondin, WT, wild type

## Abstract

**Background & Aims:**

Notch signaling maintains intestinal stem cells (ISCs). When ISCs exit the niche, Notch signaling among early progenitor cells at position +4/5 regulates their specification toward secretory vs enterocyte lineages (binary fate). The transcription factor ATOH1 is repressed by Notch in ISCs; its de-repression, when Notch is inactivated, drives progenitor cells to differentiate along the secretory lineage. However, it is not clear what promotes transition of ISCs to progenitors and how this fate decision is established.

**Methods:**

We sorted cells from Lgr5-GFP knockin intestines from mice and characterized gene expression patterns. We analyzed Notch regulation by examining expression profiles (by quantitative reverse transcription polymerase chain reaction and RNAscope) of small intestinal organoids incubated with the Notch inhibitor DAPT, intestine tissues from mice given injections of the γ-secretase inhibitor dibenzazepine, and mice with intestine-specific disruption of Rbpj. We analyzed intestine tissues from mice with disruption of the RUNX1 translocation partner 1 gene (Runx1t1, also called Mtg8) or CBFA2/RUNX1 partner transcriptional co-repressor 3 (Cbfa2t3, also called Mtg16), and derived their organoids, by histology, immunohistochemistry, and RNA sequencing (RNA-seq). We performed chromatin immunoprecipitation and sequencing analyses of intestinal crypts to identify genes regulated by MTG16.

**Results:**

The transcription co-repressors MTG8 and MTG16 were highly expressed by +4/5 early progenitors, compared with other cells along crypt-villus axis. Expression of MTG8 and MTG16 were repressed by Notch signaling via ATOH1 in organoids and intestine tissues from mice. MTG8- and MTG16-knockout intestines had increased crypt hyperproliferation and expansion of ISCs, but enterocyte differentiation was impaired, based on loss of enterocyte markers and functions. Chromatin immunoprecipitation and sequencing analyses showed that MTG16 bound to promoters of genes that are specifically expressed by stem cells (such as Lgr5 and Ascl2) and repressed their transcription. MTG16 also bound to previously reported enhancer regions of genes regulated by ATOH1, including genes that encode Delta-like canonical Notch ligand and other secretory-specific transcription factors.

**Conclusions:**

In intestine tissues of mice and human intestinal organoids, MTG8 and MTG16 repress transcription in the earliest progenitor cells to promote exit of ISCs from their niche (niche exit) and control the binary fate decision (secretory vs enterocyte lineage) by repressing genes regulated by ATOH1.

## Graphical abstract

**Figure:**
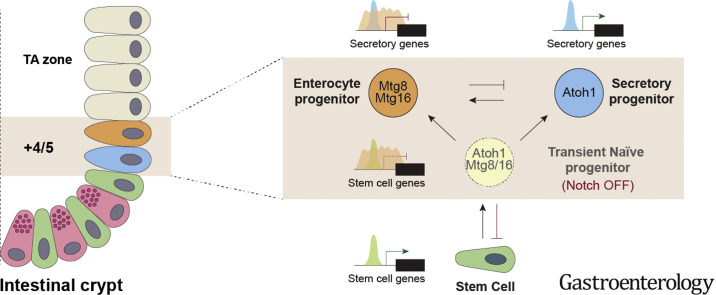

**What You Need to Know**

**Background and Context**

Notch signaling maintains intestinal stem cells (ISCs) and determines whether early progenitor cells develop along the secretory or enterocyte lineage. The transcription factor ATOH1 is repressed by Notch in ISCs; its de-repression causes precursor cells to differentiate along the secretory lineage.

**New Findings**

In the intestine, MTG8 and MTG16 are repressed by Notch signaling indirectly, via ATOH1; this promotes exit of ISCs from their niche and regulates progenitor lineage specification, by repressing ATOH1-target genes.

**Limitations**

MTG8 null mice died at birth. Studies of mice with intestine-specific knockout of MTG8 are needed to determine phenotypes of adult intestine.

**Impact**

MTG8 and MTG16 are chromatin modulators that regulate differentiation of ISCs into secretory vs enterocyte lineages.

The intestinal epithelium renews every 5 days, a process that is driven by the intestinal stem cells (ISCs) located at the crypt base. ISCs divide and give rise to early progenitor populations at the +4/5 cell position, where lineage specifications take place (Extended Data Supplementary Figure 1).^1^^,^^2^ Notch signaling plays a key role in lineage commitment and plasticity. Activation of Notch drives enterocyte differentiation, while Notch inactivation de-represses the transcription factor ATOH1, a master regulator of all secretory lineages: Paneth, goblet, and enteroendocrine cells.^3^, ^4^, ^5^, ^6^ This binary fate decision is believed to be driven by the emerging expression of the Notch ligand Delta-like (Dll) family on early secretory progenitors, which activates Notch in surrounding progenitor cells. This instructs these “naïve” neighbors to take the opposite (enterocyte) fate. This process is termed “lateral inhibition” and is proposed to be under ATOH1 regulation.^7^, ^8^, ^9^, ^10^ Dll1+ secretory progenitors exert plasticity, that is, they can revert to stem cells on stem cell loss.^11^ Although the signaling pathways regulating ISC fate are well-defined, the underlying mechanism of how stem cells commit to differentiation and undergo the subsequent binary fate decision remains largely uncharacterized. Importantly, transcriptional control and molecular markers of enterocyte progenitors remain largely undefined. Very recent studies propose that chromatin accessibility plays a crucial role in fate decisions and plasticity at the early progenitor stage.^8^^,^^12^^,^^13^

**Figure 1:**
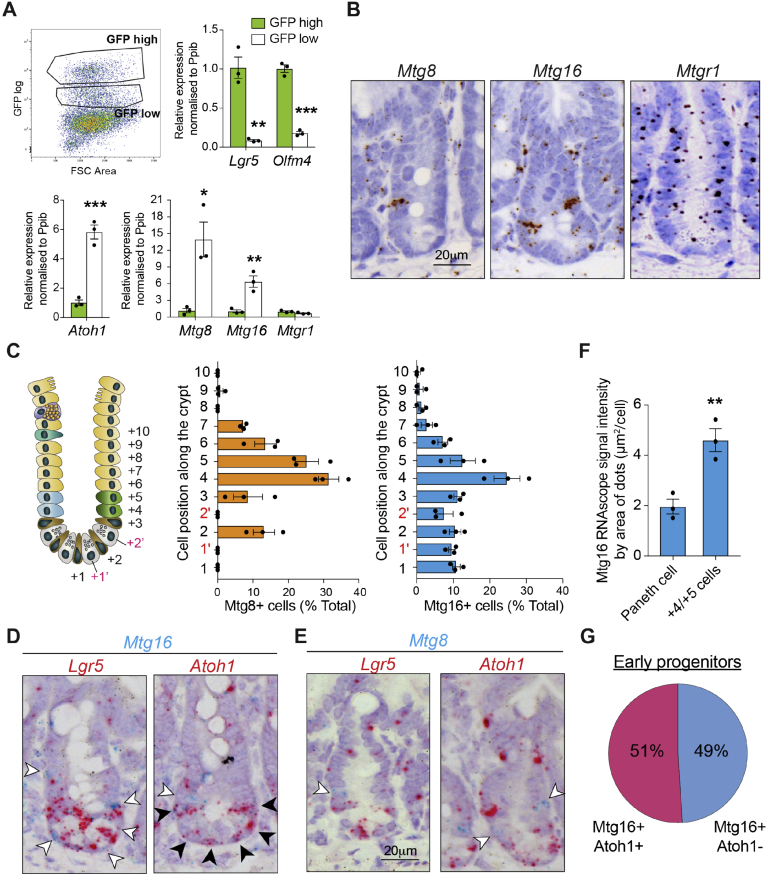
Expression of *Mtg8* and *Mtg16* in the +4/5 early progenitor cells. (*A*) FACS isolation of GFP-high and GFP-low cells from the *Lgr5-EGFP-IRES-CreERT2* intestinal crypts. qRT-PCR analysis of the indicated genes in the 2 populations. Data represent mean ± SEM from biologically independent animals (n = 3). ∗*P* < .05, ∗∗*P* < .01, ∗∗∗*P* < .001, 2-sided *t*-test. (*B*) RNAscope brown staining of *Mtg8*, *Mtg16* and *Mtgr1* in intestinal crypts from WT mice. (*C*) Quantification of *Mtg8* and *Mtg16* RNAscope staining in (*B*) along the crypt. Data represent mean ± SEM from biologically independent animals (n = 3). (*D*, *E*) RNAscope duplex staining of *Mtg16* (*D*) or *Mtg8* (*E*) (*blue*) with *Atoh1* or *Lgr5* (red) in WT intestinal crypts. *Empty arrows* indicate exclusive staining, *black arrows* indicate colocalized staining. (*F*) Quantification of *Mtg16* RNAscope signal (*area of dots*) in Paneth cells and progenitor cells. Data represent mean ± SEM from biologically independent animals (n = 3). ∗∗*P* < .01, 2-sided *t*-test. (*G*) Quantification of *Mtg16*+*Atoh1+* and *Mtg16*+*Atoh1−* cell populations in early progenitors (+3–5 positions) from the RNAscope staining in (*D*). Scale bars, 20 μm.

To delineate the early stem cell–daughter cell transition, we studied transcriptional control directly at the earliest progenitor cell at the +4/5 position on niche exit. In this study, we identified 2 transcriptional co-repressor homologues, MTG8 and MTG16, that are expressed in these early progenitors. MTG8 and MTG16 were repressed by Notch signaling both in ex vivo organoids and in vivo. We further showed that the 2 co-repressors play central roles in early fate decision of ISCs by repressing the stem cell gene expression program and *Dll* expression for lateral inhibition. Previous studies have demonstrated that MTG8 and MTG16 recruit chromatin-modifying enzymes for transcriptional regulation.^14^ Together, our findings indicate a critical role for MTG8 and MTG16 in niche exit and in early fate decision of ISCs by regulating chromatin accessibility of the target genes.

## Materials and Methods

Please refer to the online Supplementary Materials for detailed additional Methods.

### Animals and Drug Administration

All animal regulated procedures were carried out according to Project License constraints (PEF3478B3) and Home Office guidelines and regulations. *Lgr5-EGFP-IRES-CreERT2*^15^ mice were used for FACS sorting experiments. *Rbpj*^*fl/fl*^ mice^16^ were crossed with *VillinCreER*^17^ mice for inducible intestinal-specific deletion. *Mtg16*^*−/−*^, *Mtg8*^*−/−*^*;* and *Mtgr1*^*−/−*^ mice were kind gift from Scott W. Hiebert. *Lgr5DTR-EGFP* mice (kind gift from Genentech, hereafter named as *Lgr5-GFP* mice because only the green fluorescent protein [GFP] reporter element was used in this study) were crossed with *Mtg16−/−* mice to generate *Mtg16−/−*; *Lgr5-GFP* animals. *VillinCreER; Rbpj*^*fl/fl*^ or *VillinCreER* animals were injected with tamoxifen (T5648, Sigma-Aldrich) intraperitoneally at 1.6 mg per 10g of mice and collected at the indicated time points. For proliferation analysis, animals were injected intraperitoneally (IP) with 30 mg/kg EdU (E10187, Molecular Probes) 2 hours before tissue collection. EdU treatment in newborn pups was performed the same as in adults except that the pups were culled 20 minutes after injection. Dibenzazepine (4489 DBZ; Tocris, Bristol, UK) was administered to wild-type (WT) animals as described previously.^8^ Briefly, mice were injected IP twice the same day, 6 hours apart, with a dose of 100 μmol/kg DBZ suspended in 0.5% (wt/vol) hydroxypropylmethyl-cellulose (94378, Methocel E4M; Sigma-Aldrich, St Louis, MO) and 0.1% (wt/vol) Tween 80 (P1754; Sigma-Aldrich) in water or only the vehicle as a control. Mice were collected at the indicated time points after the first injection.

### Statistical Analysis

Results are expressed as mean ± standard error of the mean (∗*P* < .05, ∗∗*P* < .01, ∗∗∗*P* < .001). Statistical significance of mean values was assessed using unpaired Student *t*-test or analysis of variance, 1- or 2-way, followed by Tukey’s or Sidak’s Multiple Comparison Post-test respectively. The corresponding number of N and experiments are indicated in the figure legends. Statistics were performed using GraphPad Prism 7 software (La Jolla, CA).

## Results

### Expression of Mtg8 and Mtg16 in a Subpopulation of +4/5 Cells of Intestinal Crypt

To investigate the transcriptional regulation of stem cell fate and early lineage commitment at the +4/5 progenitor stage, we analyzed the expression profile of sorted LGR5-GFP cells (GSE36497).^18^ Rather than focusing on the GFP-high (LGR5-GFP 5+) stem cell population, we examined the GFP-low (LGR5-GFP 2+, 3+, and 4+) fractions that represent immediate daughter cells. This allowed us to identify +4/5 cell-enriched genes in the absence of a specific molecular marker. Hierarchical clustering analysis revealed 525 genes that were enriched in GFP-low populations (Supplementary Figure 1*B* and Supplementary Table 1). These included *Atoh1*, *Dll1,* and *Dll4* that have previously been reported to be expressed in the early secretory progenitors.^8^^,^^11^^,^^19^ Among the 525 genes, we further screened for transcription factors that were enriched in LGR5-GFP-low and absent in LGR5-GFP-high stem cell populations. This resulted in the identification of the 2 related transcriptional regulators *Mtg8* and *Mtg16*.

MTG8, MTG16, and MTGR1 (also known as RUNX1T1, CBFA2T3, and CBFA2T2, respectively) are transcriptional co-repressors that comprise the myeloid translocating gene (MTG) family.^14^^,^^20^ Quantitative reverse transcription polymerase chain reaction (qRT-PCR) confirmed the enriched expression of *Mtg8* and *Mtg16* in LGR5-GFP-low early progenitors in the crypts, similar to *Atoh1* (Figure 1*A* and Supplementary Figure 1*C*). RNAscope analysis further demonstrated enriched expression of *Mtg8* and *Mtg16* at +4/5 cell positions directly above the LGR5+ ISC compartment (Figure 1*B–D*), whereas *Mtgr1* was expressed throughout the crypt (Figure 1*A* and *B*). Quantification of the RNAscope signal confirmed that most *Mtg8*+ cells were present at positions 4 and 5 (31.5% and 25.3%, respectively), whereas *Mtg16*+ cells were distributed throughout the lower crypt with a peak frequency at positions 4 and 5 (24.7% and 12.6%, respectively) (Figure 1*C*). RNAscope co-staining further revealed that expression of *Mtg16* was mostly exclusive with *Lgr5* but colocalized with *Atoh1* at the crypt bottom, indicating that Mtg16 is also expressed by Paneth cells (Figure 1*D*). It is worth noting that the RNAscope signal of *Mtg16* was significantly stronger at positions 4 and 5 than in Paneth cells (Figure 1*F*, Supplementary Figure 1*D*), indicating that *Mtg16* is indeed enriched in early crypt progenitors. Expression of *Mtg8* was mostly exclusive from *Atoh1* (Figure 1*E*), whereas *Mtg16* was expressed in both *Atoh1+ and Atoh1−* populations in the early progenitors in roughly equal proportions (Figure 1*D* and *G*). *Mtg8* was also found to colocalize with *Mtg16* (Supplementary Figure 1*E*). In addition, co-staining of *Mtg16* and Muc2 showed that *Mtg16* was also expressed in a small subset of goblet cells that were mainly localized at the crypt-villus junction (red arrows in Supplementary Figure 1*F*). We also observed stromal expression of *Mtg16* in the intestine (Figure 1*B*, white arrows in Supplementary Figure 1*F*). Because MTG16 is required for hematopoietic progenitor cell fate decision,^21^ it is highly likely that the mesenchymal expression of *Mtg16* is localized to hematopoietic cells. Taken together, our data suggest that a subpopulation of +4/5 cells express *Mtg8/16* and are negative for *Atoh1*. Of note, *Mtg8* transcript levels were very low in abundance throughout the intestinal tissue, suggesting that its expression might be transient and dynamic under strict regulation at the +4/5 cells.

### Mtg8 and Mtg16 Are Negatively Regulated by Notch Signaling

**Figure 2:**
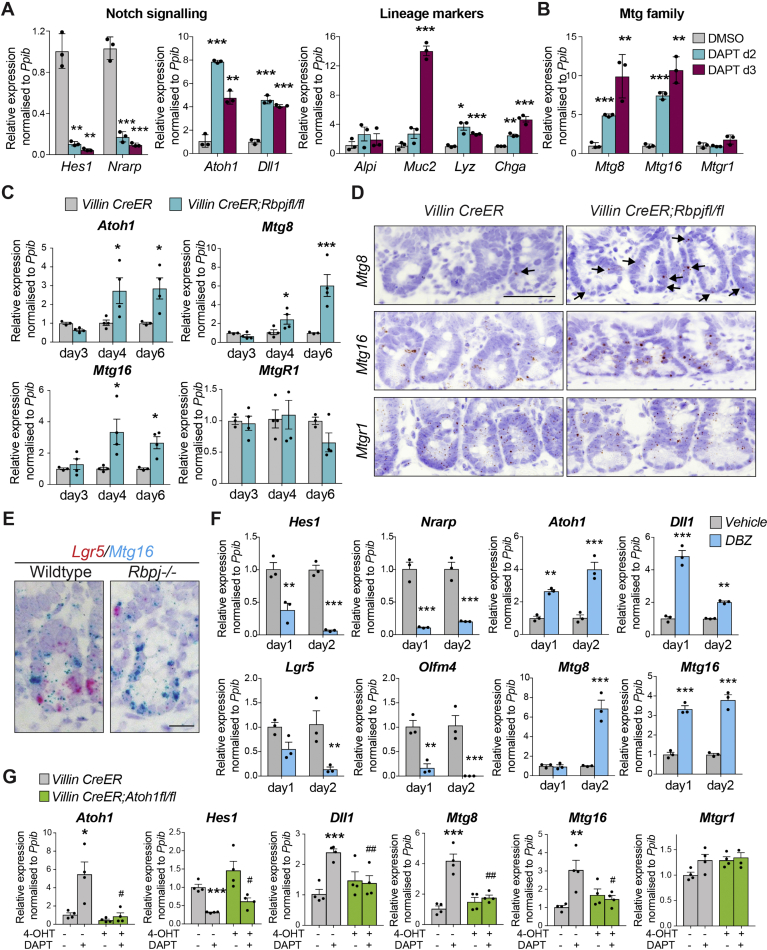
*Mtg8* and *Mtg16* are regulated by Notch signaling. (*A*, *B*) qRT-PCR analysis of WT mouse intestinal organoids treated with Notch inhibitor DAPT for 2 and 3 days. Data represent mean ± SEM from biologically independent small intestinal organoid isolations (n = 3). The experiment was performed twice. ∗*P* < .05, ∗∗*P* < .01, ∗∗∗*P* < .001, 2-sided *t*-test. (*C*) qRT-PCR analysis of intestinal epithelium from *Villin CreER* and *Villin CreER;Rbpjfl/fl* mice collected at indicated days after tamoxifen induction. Data represent mean ± SEM from biologically independent animals (n = 4 per group). Three independent experiments were performed. ∗*P* < .05, ∗∗*P* < .01, ∗∗∗*P* < .001, 2-way analysis of variance (ANOVA). (*D*) RNAscope brown staining of *Mtg8*, *Mtg16,* and *Mtgr1* in intestinal tissue obtained from *Villin CreER* and *Villin CreER;Rbpjfl/fl* mice collected at day 4 post-tamoxifen induction. *Arrows* indicate Mtg8+ cells. Scale bars, 50 μm. (*E*) RNAscope duplex staining of *Mtg16* (*blue*) and *Lgr5* (*red*) in intestinal tissues of the indicated genotypes at day 6 post-tamoxifen induction. (*F*) qRT-PCR analysis of intestinal epithelium from WT mice collected at the indicated days after DBZ or vehicle treatment. Data represent mean ± SEM from biologically independent animals (n = 3 per group). ∗*P* < .05, ∗∗*P* < .01, ∗∗∗*P* < .001, 2-way ANOVA. (*G*) qRT-PCR analysis of DAPT-treated *Villin CreER* and *Villin CreER;Atoh1fl/fl* organoids induced with 4-OHT. Data represent mean ± SEM. The experiment was performed 4 times. ∗*P* < .05, ∗∗*P* < .01, ∗∗∗*P* < .001, compared with untreated control group; #*P* < .05, ##*P* < .01, compared with DAPT-treated control group, 2-sided *t*-test.

Because *Mtg16* expression partially overlaps with *Atoh1* expression in +4/5 cells, we asked whether the co-repressors are regulated by Notch signaling. Mouse small intestinal organoids were treated with the Notch inhibitor DAPT followed by qRT-PCR analysis. As expected, DAPT-treated organoids showed significant suppression of Notch signaling and de-repression of *Atoh1* and *Dll1*, while secretory lineage markers were upregulated (Figure 2*A*). Remarkably, both *Mtg8* and *Mtg16*, but not *Mtgr1*, were significantly upregulated upon DAPT treatment (Figure 2*B*). We observed similarly upregulated expression of *MTG8* and *MTG16* in DAPT-treated human intestinal organoids, indicating that the Notch-controlled expression of the 2 genes is conserved in human (Supplementary Figure 2*A*).

To confirm the Notch regulation in vivo, we depleted the Notch downstream transcription factor *Rbpj* for 3 to 6 days. Loss of *Rbpj* resulted in a progressive drift of differentiation toward the secretory lineage, concurrent with increased *Atoh1* expression (Figure 2*C*; Supplementary Figure 2*B* and *C*). Consistent with the organoid data, qRT-PCR analysis showed robust upregulation of *Mtg8* and *Mtg16* in the intestinal crypts upon *Rbpj* loss in vivo, while *Mtgr1* expression was unchanged (Figure 2*C*). Interestingly, expression of *Mtg8* and *Mtg16* was also significantly increased at day 4 post-*Rbpj* deletion in vivo, when most of the differentiation markers remained unchanged (Figure 2*C* and Supplementary Figure 2*C* and *D*). RNAscope staining confirmed upregulation of *Mtg8* and *Mtg16*, and loss of the ISC marker *Lgr5* upon *Rbpj* deletion (Figure 2*D* and *E*). We further validated the findings by treating WT animals with the γ-secretase inhibitor, DBZ, as an alternative Notch inactivation model, which again resulted in a progressive shift toward the secretory lineage (Supplementary Figure 2*E*). Similar to the *Rbpj* deletion data, expression of *Atoh1*, *Mtg8,* and *Mtg16* was significantly upregulated immediately after treatment (1–2 days) (Figure 2*F* and Supplementary Figure 2*F*), confirming that MTG8 and 16 are repressed by Notch signaling. Previous data have suggested regional differences in Notch signaling in the intestine, where proximal duodenum shows higher Notch signaling than distal parts of ileum.^22^ In agreement, higher expression of *Atoh1* and *Mtg16* was observed in the distal ileum (Supplementary Figure 2*G*).

Because the secretory progenitor marker ATOH1 is also repressed by Notch signaling in +4/5 cells, we asked whether the Notch-regulated *Mtg8* and *Mtg16* expression is dependent on ATOH1 using *Atoh1 floxed* organoids. Indeed, the DAPT-induced expression of *Mtg8* and *Mtg16* was abrogated on *Atoh1* deletion, indicating that *Mtg8* and *Mtg16* expression is mediated by Atoh1 (Figure 2*G*). Furthermore, ectopic expression of ATOH1 in HEK293T cells was able to induce *MTG8* and *MTG16* expression (Supplementary Figure 2*H*), indicating that the Atoh1-mediated MTG expression is independent of change of cell fate. To verify the hierarchical regulation of ATOH1 and MTG8/16, we further examined their expression dynamics in a short time-course DAPT treatment of organoids. The results demonstrated that *Atoh1* was significantly upregulated 12 hours after DAPT induction, whereas *Mtg16* was only upregulated 15 hours after induction (Supplementary Figure 2*I*). *Mtg8* remained unchanged during the first 16 hours of DAPT treatment. The data suggest that the expression of *Mtg8* and *Mtg16* is likely to be driven by Atoh1 at different dynamics. We conclude that MTG8 and MTG16, but not MTGR1, are repressed by Notch signaling indirectly via ATOH1 in the intestine.

### Loss of Mtg8 and Mtg16 Induces Hyperproliferation and Expansion of ISCs

**Figure 3:**
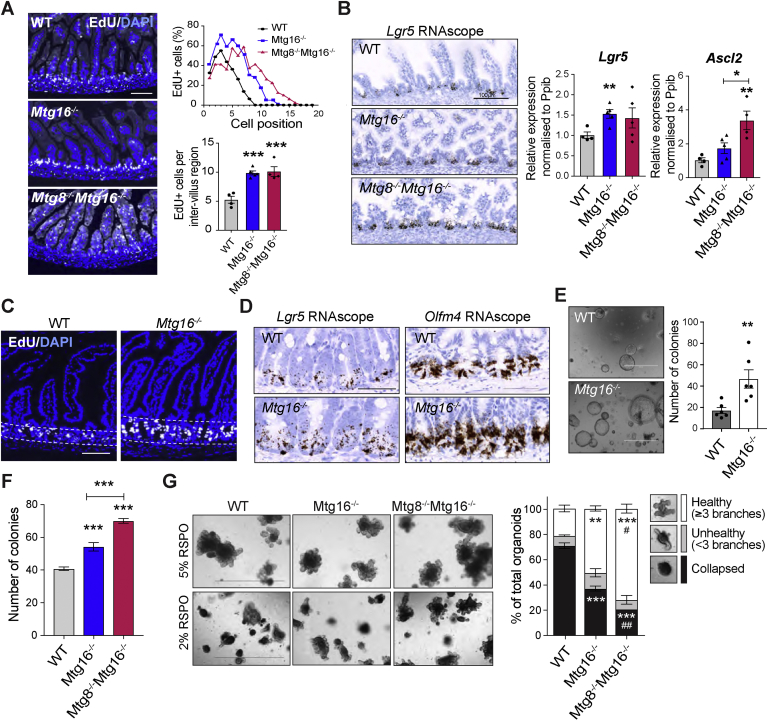
Loss of *Mtg8* and *Mtg16* increases ISC numbers and proliferation. Intestinal tissues were collected from newborn (P0) (n = 4–5 for each genotype) (*A*, *B*) or adult mice (n = 3–6 mice per group) (*C–E*) for analysis. (*A*) EdU staining showing increased proliferation in *Mtg16−/−* and *Mtg8−/− Mtg16−/−* animals compared with WT. Graphs showing EdU+ cells distribution along the crypt and quantitation of EdU+ cells per inter-villus region in WT, *Mtg16−/−* and *Mtg8−/−Mtg16−/−* intestine. Data represent mean ± SEM of 3 independent experiments. At least 10 representative crypts per animal have been analyzed. (*B*) *Lgr5* RNAscope staining and qRT-PCR showing increased ISC gene expression in newborn *Mtg16−/−* and *Mtg8−/−Mtg16−/−* tissues. Data represent mean ± SEM of 3 independent experiments (n = 4 per group). ∗*P* < .05, ∗∗*P* < .01, ∗∗∗*P* < .001, 1-way analysis of variance (ANOVA). (*C*) EdU staining in WT and *Mtg16−/−* adult intestine. (*D*) *Lgr5* and *Olfm4* RNAscope brown staining in small intestinal tissue from WT and *Mtg16−/−* adult mice. (*E*) Colony formation assay of small intestine organoids isolated from WT and *Mtg16−/−* adult mice. Data represent mean ± SEM of 2 independent experiments (n = 6 mice per group). ∗*P* < .05, ∗∗*P* < .01, ∗∗∗*P* < .001, 2-sided *t*-test. Scale bars, 100 μm (*B–D*), 1000 μm (*E*). (*F*) Colony formation assay of small intestine organoids derived from WT, *Mtg16−/−* and *Mtg16−/−Mtg8−/−* newborn pups. Data represent mean ± SEM of 3 independent experiments (n = 2–4 mice per group). ∗∗∗*P* < .001, 1-way ANOVA. (*G*) Representative images showing newborn organoids of the indicated genotypes cultured in 5% or 2% RSPO conditions for 3 to 4 days. Scale bar, 1000 μm. *Right*, quantification of the organoid health status maintained in 2% of RSPO condition. Data represent mean ± SEM of 2 independent experiments (n = 2–4 mice per group). ∗∗*P* < .01, ∗∗∗*P* < .001 compared with WT, 1-way ANOVA. #*P* < .05, ##*P* < .01 compared to *Mtg16−/−*, 1-way ANOVA.

The Notch-regulated expression of *Mtg8* and *Mtg16* in +4/5 cells led us to investigate whether the co-repressors play a role in ISC fate decision. We analyzed the *Mtg8−/−* and *Mtg16−/−* animals. *Mtg16−/−* mice were healthy and viable, whereas *Mtg8−/−* and double knockout (DKO) animals died shortly after birth, in accordance with previous findings.^21^^,^^23^ We then proceeded to analyze *Mtg16−/−* and DKO newborn pups. The intestine obtained from DKO pups was significantly shorter than from WT, which was consistent with the Mtg8-null phenotype (Supplementary Figure 3*A*).^23^ Both *Mtg16−/−* and DKO animals showed significantly increased proliferation in the inter-villus regions that would later give rise to crypts (Figure 3*A* and Supplementary Figure 3*B*). Notably, EdU+ cells were also detected in the villi of the DKO intestine, indicating that epithelial cell proliferation was extended beyond inter-villus regions to the villi. Next, we analyzed whether the increase in the inter-villus epithelial cell proliferation is accompanied by upregulated ISC gene expression. RNAscope and qRT-PCR demonstrated that the ISC marker *Lgr5* and its transcriptional activator *Ascl2* were both significantly upregulated in the mutants (Figure 3*B*). Similarly, significant increases in crypt proliferation and ISC markers (*Lgr5* and *Olfm4*) were also observed in *Mtg16−/−* adult intestine (Figure 3*C* and *D*; Supplementary Figure 3 *C* and *D*). Co-staining of *Lgr5* and *Atoh1* confirmed significantly increased *Lgr5* expression in the trans-amplifying region above the Atoh1+ progenitors in the mutant intestines (Supplementary Figure 3*E*). We further generated *Mtg16−/−;Lgr5-GFP* mice to label the endogenous LGR5+ ISCs. In agreement with the RNAscope observations, an increased number of GFP+ ISCs were detected in the *Mtg16−/−* adult intestine, confirming the ISC expansion phenotype (Supplementary Figure 3*F*).

To test whether the stem cell–repressive role of MTG16 is cell-intrinsic, we further examined the colony formation capacity of WT and mutant organoids ex vivo in the absence of any stromal niche. We confirmed that *Mtg16−/−* organoids grew faster than the WT controls, suggesting that the stem cell–repressive role of MTG16 is indeed cell-intrinsic (Figure 3*E*). Similarly, organoids derived from *Mtg16−/−* and DKO newborn intestine also grew significantly faster than the WT counterparts (Figure 3*F* and Supplementary Figure 3*G*). We further challenged the organoids by reducing the Wnt agonist R-spondin (RSPO) concentration. Neither WT nor MTG mutant organoids survived in the absence of RSPO. However, *Mtg16−/−* and DKO organoids grew significantly better in the low-RSPO (2%) condition with fewer collapsed organoids and more healthy branching organoids compared with WT (Figure 3*G*). The results suggest that MTG KO organoids have a growth advantage in the low-RSPO condition but are still dependent on exogenous Wnt signaling for ISC survival. Because MTG16 is expressed in Paneth cells, we asked whether the increase in crypt proliferation and stem cell markers in *Mtg16−/−* intestine is caused by dedifferentiation of Paneth cells, which has recently been reported to occur on injury.^24^ However, immunostaining of WT and *Mtg16*−/− tissue did not show any colocalization of Paneth cell marker lysozyme and proliferation marker Ki67 (Supplementary Figure 3*H*), indicating that the increase in stem cell gene expression on MTG16 loss is caused by stem cell de-repression in the early progenitors rather than Paneth cell plasticity. Together, we tentatively conclude that MTG8 and MTG16 regulate niche exit at the +4/5 cells by repressing the ISC fate and proliferation.

### Mtg8 and Mtg16 Deletion Impairs Intestinal Lineage Specification

**Figure 4:**
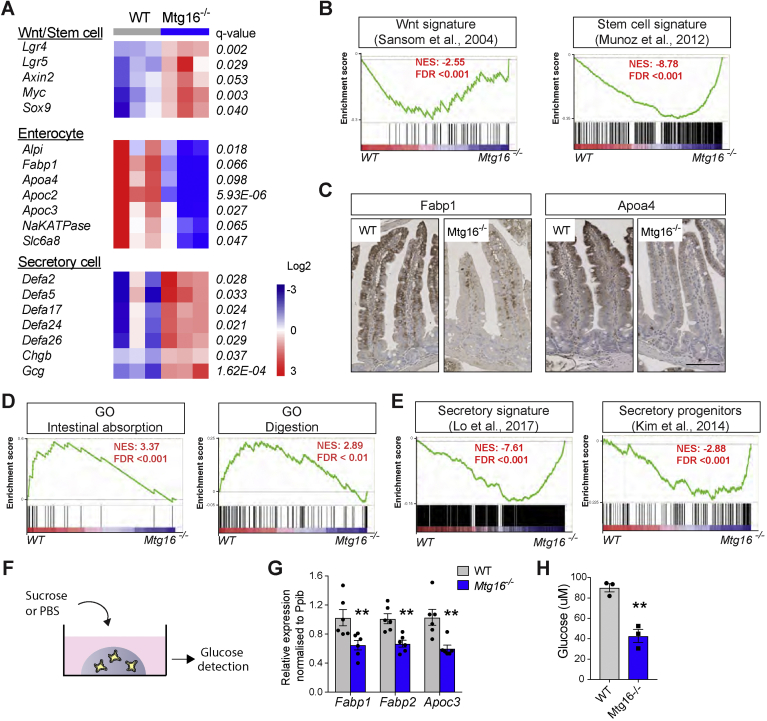
*Mtg8* and *Mtg16* deletion impairs intestinal lineage specification. Intestinal tissues were collected from newborn (P0) (n = 4–5 for each genotype) (*A*) Heatmap showing genes differentially expressed in WT and *Mtg16−/−* intestine. (*B*, *D*, *E*) Gene Set Enrichment Analysis (GSEA) probing (*B*) Wnt/Stem cell signature genes, (*D*) intestinal absorption and digestion, and (*E*) secretory signature genes. (*C*) FAPB1 and APOA4 immunostaining in adult WT and *Mtg16−/−* intestinal tissue. (*F*) Scheme showing the disaccharidase assay performed in organoids. (*G*) qRT-PCR of mature enterocyte markers in adult WT and *Mtg16*−/− organoids. (*H*) Glucose levels detected in the supernatant of intestinal organoids of the indicated genotypes after 1-hour sucrose incubation. ∗*P* < .05, ∗∗*P* < .01, ∗∗∗*P* < .001, 2-sided *t*-test.

MTGR1 has previously been shown to be required for intestinal secretory cell differentiation in adult tissue.^25^ We therefore asked whether loss of MTG8/16 would alter lineage selection in the intestine. Because intestinal differentiation is incomplete in newborn animals, we decided to focus on analyzing *Mtg16−/−* adult intestine. Reduced goblet cell numbers (AB-PAS) were observed in *Mtg16−/−* adult intestine (Supplementary Figure 4*A* and *B*). This is in concordance with the previously reported phenotype of *Mtg16 null* animals.^26^ Similarly, there was a tendency toward reduction of enterocyte markers (villin and alkaline phosphatase) in the mutant intestines (Supplementary Figure 4*A*). We believe that the moderate alteration of terminal differentiation might be due to the redundant role of Mtg8. To provide a global, unbiased picture of gene expression changes in the mutant animals, we further performed transcriptional profiling on the WT and *Mtg16−/−* intestine. RNA-seq analysis revealed 478 genes that were differentially expressed upon *Mtg16* deletion (Supplementary Figure 4*C* and *D*; Supplementary Table 2). Consistent with the increased crypt proliferation observed in Figure 3, Wnt and stem cell signatures^18^^,^^27^ were both significantly upregulated upon loss of MTG16 (Figure 4*B*). Interestingly, we further observed significant reduction of enterocyte markers and upregulation of secretory markers such as Paneth cells and enteroendocrine cells in the *Mtg16* mutant intestine (Figure 4*A*). Of note, RNA-seq data did not reveal significant alteration of goblet cell markers. Comparison of various enterocyte markers with the previously published single-cell RNA-seq data (GSE92332) revealed differential expression of the markers between mature and immature enterocytes.^28^ In particular, *Alpi* expression did not distinguish between mature and immature enterocytes, while other markers such as *Apoa4*, *Fabp1* and *Fabp2* were preferentially expressed in mature enterocytes (Supplementary Figure 4*E*). Our RNA-seq data suggested that deletion of Mtg16 results in a loss of mature enterocyte markers. Indeed, a clear reduction of FABP1 and APOA4 proteins was observed in the *Mtg16−/−* intestine, indicating that loss of MTG16 inhibits enterocyte differentiation and maturation (Figure 4*C*). Gene set enrichment analysis further confirmed the loss of absorptive signatures (Figure 4*D*) and enrichment of secretory signatures^8^^,^^29^ (Figure 4*E*) in the *Mtg16−/−* intestine. To further demonstrate the functional defect of the MTG mutant intestine, we examined the disaccharidase (brush border enzyme) activity in the WT and *Mtg16−/−* organoids (Figure 4*F*). Downregulated expression of mature enterocyte markers was confirmed in *Mtg16−/−* organoids (Figure 4*G*). Consistent with our observation of impaired enterocyte differentiation *in vivo*, the disaccharidase activity of the adult *Mtg16−/−* organoid was significantly reduced when compared to WT control organoids (Figure 4*H*).

Taken together, our data support the notion that MTG16 represses stem cell proliferation and promotes enterocyte over secretory lineage differentiation. Interestingly, we also noted enrichment of chromatin remodeling and epigenetic regulatory genes in the *Mtg16* mutant intestine (Supplementary Figure 4*F*), suggesting that the co-repressor MTG16 may regulate gene expression by chromatin remodeling.

### Mtg16 Binds to ISC Signature Genes and Atoh1-targets for Niche Exit and Fate Decision

**Figure 5:**
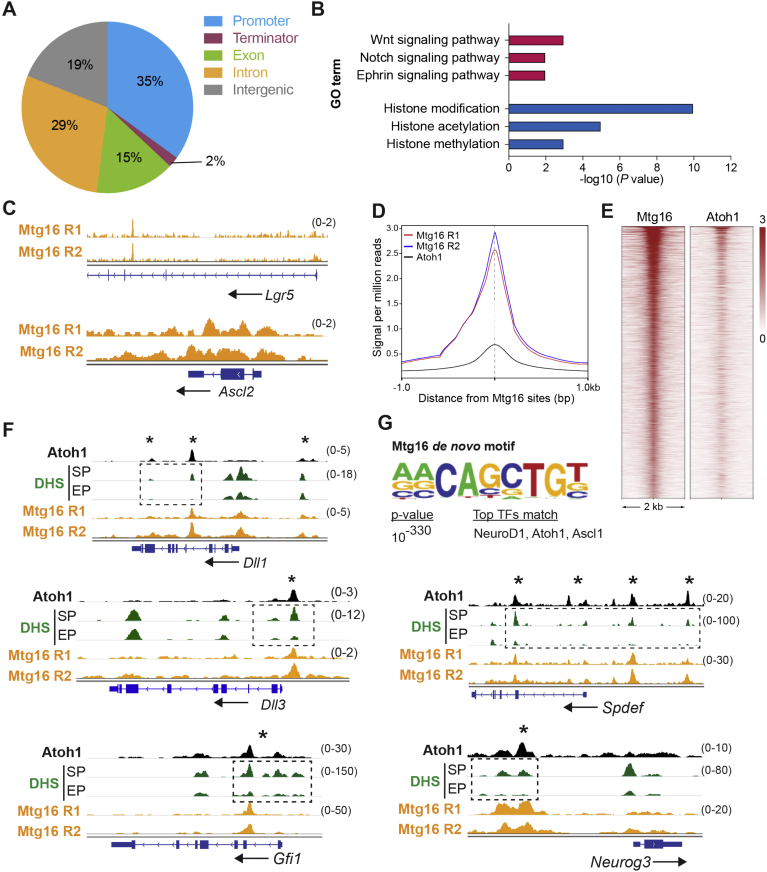
MTG16 binds to ISC- and secretory lineage-signature gene loci. (*A*) Genome-wide distribution of 7843 MTG16-binding sites. (*B*) Gene ontology analysis identified ontology terms associated significantly with MTG16 targetome, including Wnt, Notch, and Ephrin pathways, as well as in histone-modifying genes. (*C*) ChIP-seq data showing MTG16 binding signal (per million reads) to ISC gene loci (*Lgr5* and *Ascl2*). (*D*, *E*) Composite profile (*D*) and heatmap (*E*) showing striking overlap between ATOH1 and MTG16 binding sites (7843 sites). (*F*) ChIP-seq data showing MTG16 binding signal (per million reads) to previously reported ATOH1-enhancer regions^12^ (*asterisk*) of the indicated gene. Reduced levels of DHS in enterocyte progenitors (EP) versus secretory progenitors (SP) are indicated by *dotted box*. (*G*) MTG16 de novo motif matches with previously reported ATOH1 and ASCL1/2 motif.

To investigate how the co-repressors regulate ISC gene expression program and lineage selection, we then performed chromatin immunoprecipitation coupled-deep sequencing (ChIP-seq) to identify the MTG targetome. To capture the physiological binding targets in vivo, intestinal crypts were isolated 4 days after *Rbpj*-depletion to enhance endogenous MTG16 expression, while most of the differentiation markers remained unaltered (Figure 2*C* and *D* and Supplementary Figure 2*C* and *D*). MTG16 ChIP-seq identified 7843 reproducible binding sites (Figure 5*A*, Supplementary Figure 5*A* and Supplementary Table 3). Gene ontology analysis of MTG16 targets revealed enrichment of genes associated with Wnt and Notch signaling, as well as histone-modifying genes (Figure 5*B* and Supplementary Table 4). Comparison between the ChIP-seq and RNA-seq data showed that 35% of the genes differentially expressed upon *Mtg16* deletion harbored MTG16-binding sites within 5kb of the transcription start site (Supplementary Figure 5*B*), where the odds of genes being differential were observed to be increased by a factor of 2.4 (*P* < 2e-16, hypergeometric test). In particular, we observed clear MTG16-binding signals over the key ISC genes *Lgr5* and *Ascl2* (Figure 5*C*). These sites coincided with the previously reported regulatory regions in these genes.^30^^,^^31^ MTG16 also bound to the promoter regions of other Wnt targets such as *Axin2, Myc,* and *Sox9*, suggesting that MTG16 represses ISC signature genes and Wnt targets through direct binding to their regulatory sequences (Supplementary Figure 5*C*). This result was consistent with our observation that *Lgr5* and *Ascl2* are upregulated on MTG8 and MTG16 loss (Figure 3*B* and *D*).

Because MTG8 and MTG16 are repressed by Notch signaling in +4/5 cells, we asked whether they play a role in lineage selection, similar to ATOH1. We compared our MTG16 ChIP-seq data with the previously reported ATOH1 ChIP-seq and DNase I hypersensitivity (DHS, a measure of chromatin accessibility) data on secretory- or enterocyte progenitors.^8^ A striking overlap (84.08%) between MTG16 and ATOH1 binding sites was observed between the 2 datasets, suggesting that MTG16 may also be involved in fate decision (Figure 5*D* and *E*).

ATOH1 has previously been reported to drive lateral inhibition and to set the secretory fate by regulating expression of the *Dll* Notch ligands.^8^^,^^29^ We analyzed the ChIP-seq profiles of the Notch ligands *Dll1 and Dll3*. Remarkably, we found that MTG16 bound to the previously reported ATOH1-enhancer regions of both *Dll1 and Dll3* (Figure 5*F*). Interestingly, loss of or reduced levels of DHS were observed in enterocyte progenitors compared with secretory progenitors at the regions where MTG16- and ATOH1-binding overlapped (Figure 5*F*). These results suggest that MTG16 binds to the ATOH1-bound loci to reduce chromatin accessibility of *Dll* genes in enterocyte progenitors for lateral inhibition and early fate decision. Similar to *Dll* ligands, MTG16 also occupied most of the reported ATOH1-binding sites in all secretory signature genes including *Spdef*, *Gfi1,* and *Neurog3* with reduced DHS levels in enterocyte progenitors (Figure 5*F*). Given that MTG proteins repress gene transcription by recruiting various chromatin-modifying enzymes (eg, histone deacetylases),^14^ we propose that MTG8/16 regulate lateral inhibition and binary fate decisions of +4/5 progenitors by repressing ATOH1-mediated *Dll* ligands and secretory signature gene transcription. Indeed, de novo motif analysis identified an MTG16 consensus motif that was matched to the reported ATOH1 motif, suggesting that MTG16 occupies ATOH1-bound enhancers to regulate lineage specification (Figure 5*G* and Supplementary Table 5). Co-immunoprecipitation analysis further confirmed the physical binding of ATOH1 with both MTG8 and MTG16 (Supplementary Figure 5*D*). Consistently, ATOH1-mediated *DLL1* expression was significantly downregulated by MTG8 or MTG16 expression (Supplementary Figure 5*E*). In addition to ATOH1, MTG family members have previously been shown to interact with TCF4 for transcriptional suppression.^32^ Together, our findings support the notion that the MTG co-repressors bind to the transcription factors TCF4/β-catenin and ATOH1 to repress the stem cell program and *Dll* expression for lateral inhibition. We further noted that MTG16 bound strongly to its own locus as well as to the promoter regions of *Mtgr1* and *Atoh1* (Supplementary Figure 5*F*), whereas MTG16 has recently been reported as an ATOH1 target.^29^ The data imply that ATOH1 and the MTG family together contribute to a “cross-over” feedback loop in +4/5 cells to regulate rapid, dynamic fate decisions.

## Discussion

**Figure 6:**
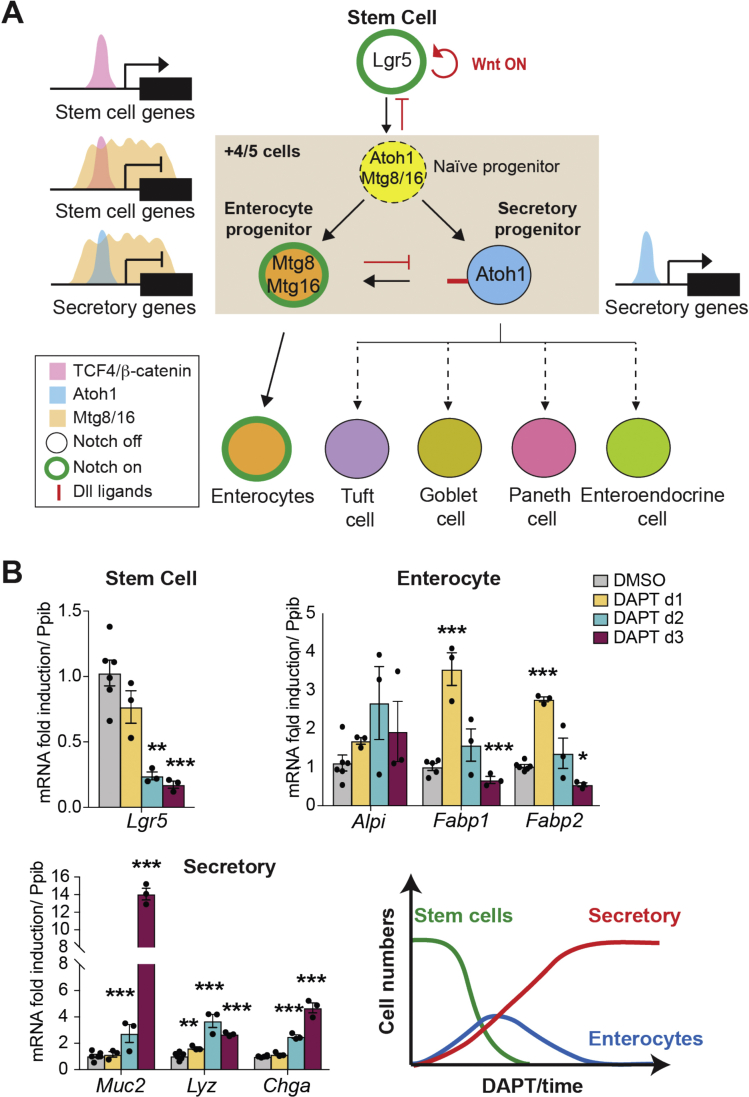
Proposed model for intestinal stem cell hierarchy. (*A*) Updated ISC fate model. See text for details. (*B*) qRT-PCR analysis of the indicated genes after 1, 2, or 3 days of DAPT treatment. On the *right*, illustration of expression kinetics of the stem cell, secretory and enterocyte markers upon time-course Notch inhibition. ∗*P* < .05, ∗∗*P* < .01, ∗∗∗*P* < .001, 2-sided *t*-test.

Extensive studies in the past have focused on characterizing the signaling pathways regulating ISCs, yet it has remained elusive how the tightly regulated ISC fate remains restricted to a fixed number of proliferative cells at the crypt base. Paneth cells have been shown to constitute the essential niche to define ISC identity,^9^^,^^33^, ^34^, ^35^ yet functional ISCs can be maintained upon Paneth cell ablation.^36^^,^^37^ Therefore, it remains unclear how Paneth cells contribute to ISC homeostasis. The undifferentiated cells immediately above the ISC compartment (+4/5 progenitors) are heterogeneous in terms of marker gene expression. ATOH1 marks a subpopulation of +4/5 cells that have entered the secretory lineage differentiation and mediate lateral inhibition,^3^^,^^8^ while molecular markers of the remaining +4/5 progenitors entering the absorptive enterocyte differentiation have not yet been identified. Here we report that the Notch-repressed transcriptional co-repressors MTG8 and MTG16 are expressed in +4/5 progenitors to switch off the stem cell expression program. Our current findings provide insights into the underlying mechanism of ISC fate decisions (Figure 6*A*). Our data support the notion that the “Notch-off” state is the first “priming” step to drive ISC-daughter cell transition by committing to transient bi-potent progenitors, which is consistent with the recently proposed “multi-lineage progenitor” population as the earliest progeny of LGR5+ stem cells.^38^ When an ISC occupies the +4/5 cell position and loses its contact with *Dll*-expressing Paneth cells (niche exit), Notch is switched off as a consequence, thereby de-repressing ATOH1, MTG8 and MTG16. The co-repressors then drive differentiation by switching off the Wnt-mediated ISC gene expression program in the immediate progenitors, leading to transient activation of the whole differentiation program. This is consistent with the data obtained from our time-course DAPT-treated organoids, where downregulation of ISC markers was accompanied by transient upregulation of both absorptive and secretory lineage markers upon early Notch inhibition (Figure 6*B*). Subsequently, ATOH1 and MTG8/16 work together in these naïve bi-potent progenitors to control lateral inhibition and binary fate decision (Figure 6*A*). Our findings uncover a novel role of MTG8/16 in promoting enterocyte differentiation by direct repression of ATOH1-mediated secretory differentiation and *Dll* ligands expression. The differential expression dynamics of *Atoh1*, *Mtg8,* and *Mtg16* and their potential negative feedback network may perhaps explain the heterogeneity within the early progenitor population. MTG16 is initially co-expressed with ATOH1 immediately after niche exit and Notch inhibition. Subsequently, MTG8 and MTG16 expression starts to dominate and repress ATOH1 expression, resulting in MTG8/16+ATOH1− cells. It is conceivable that the fate decision at these progenitors is dependent on the expression dynamics of ATOH1 and MTG. Interestingly, 2 recent studies showed direct binding of HES1 to the promoter of *Mtg16,*^39^^,^^40^ suggesting that MTG8/16 may also be actively repressed by Notch directly via HES1 at the ISCs. It is also worth noting that all *Dll* ligands are transcriptional targets of ATOH1 and MTG16, including *Dll3* that has previously been reported to function exclusively as cis-inhibition rather than trans-activation of Notch signaling.^41^ This may imply a previously underappreciated role of DLL3 in the dynamic lateral inhibition and fate decision in the early progenitors, where DLL1/4 trans-activate Notch in the neighboring cells and DLL3 inhibits Notch cell-autonomously.

Previous studies have shown that MTG16 is required for injury-induced epithelial cell survival and regeneration in the intestine.^26^^,^^42^ Interestingly, increased proliferation of *Mtg16*-depleted intestine has been demonstrated, although the underlying mechanism remained uncharacterized.^26^ More recently, increased ß-CATENIN staining has also been observed in MTG16-deleted colitis-associated tumors,^43^ suggesting a potential Wnt inhibitory role of MTG16. In the current study, we focused on characterizing the mechanistic role of MTG in normal intestinal stem cell homeostasis. Beyond the increase in crypt proliferation as previously reported, we further observed a significant increase in stem cell number in the MTG mutants. Global genomic and transcriptomic analysis further revealed that MTG16 binds to the gene loci of stem cell and Wnt signature genes for transcriptional repression. Our data on the Notch-regulated MTG expression at +4/5 progenitor cells provide mechanistic insight into how MTG regulates stem cells and the Wnt transcriptional program under normal stem cell homeostasis, which will help understand the tumor suppressive role of MTG in colorectal cancer.^44^

Regulation of chromatin accessibility has recently been reported in these highly dynamic progenitors for fate decision and plasticity.^8^^,^^12^^,^^13^ It is believed that dynamic reorganization of chromatin remodeling controls the rapid, dynamic lineage specifications of early progenitors, as well as permitting dedifferentiation of progenitors into stem cells on damage. However, the underlying mechanism of how chromatin remodeling is regulated remains unknown. The discovery of the co-repressors Mtg8/16 in the +4/5 cells offers a compelling explanation for this epigenetic regulation by recruiting various chromatin-modifying enzymes to stem cell- and lineage-specific genes for dynamic fate decisions. Controlling the expression of MTG8 and MTG16, via Notch signaling upon damage could allow the early progenitors to reacquire multipotency by de-repressing the ISC gene expression program. It is interesting to note that MTGR1 is not regulated by Notch signaling despite the previously reported role in secretory lineage differentiation.^25^ Because our ChIP-seq data revealed that MTGR1 is a transcriptional target of MTG16, it is conceivable that the MTG family function together with ATOH1 to drive fate decision via transcription activator-repressor network and chromatin remodeling. Our findings provide a direct link between Notch signaling and chromatin remodeling for ISC fate decision. Further characterization of MTG8, MTG16 and MTGR1 targetomes will help understand their transcriptional regulation of ISC fate as homodimer or heterodimer.

## Supplementary Methods

### Expression Analysis of LGR5-GFP Sorted Cells

Transcriptomic data of LGR5-GFP sorted cells was extracted from GSE36497 (Agilent array 4x44K).^1^ LGR5-GFP high (5+) represents stem cell population, while LGR5-GFP-low (4+ to 2+) represents immediate daughter cells. Given the dynamic and heterogeneous nature of the +4/5 early progenitor cells, we decided to include LGR5-GFP 2+, 3+, and 4+ cell fractions together as GFP-low progenitors for hypothesis-free unsupervised clustering analysis. Of note, the GFP-negative population that consists of differentiated cells in the villi as well as non–GFP-expressing crypt cells was not included in the analysis. We further selected genes with >2-fold difference in at least 3 arrays and performed hierarchical clustering. This resulted in 525 genes that were enriched in GFP-low populations (Supplementary Figure 1*B* and Supplementary Table 1).

### Crypts Isolation and Mouse Organoids Culture

Organoids were established from freshly isolated adult WT and *Mtg16−/−* small intestine, or P0 WT, *Mtg16−/−* and *Mtg8−/−Mtg16−/−* intestine. Tissues were incubated in cold phosphate-buffered saline (PBS) containing 2 mM EDTA for isolating epithelial crypts and cultured in Cultrex BME, Type 2 RGF PathClear (Amsbio, Abington, UK; 3533-010-02) as previously described.^2^ All freshly isolated organoids were initially cultured in IntestiCult Organoid Growth Medium (Stem Cell technologies, Vancouver, BC, Canada; #06005). For P0 intestine, organoids were cultured for at least 4 weeks before any experiments to allow maturation. The Rho kinase inhibitor Y-27632 (Sigma, Y0503) was added to the culture during first week of crypt isolation and single cell dissociation. For R-spondin (RSPO) depletion experiments, organoids were switched to basal media containing epidermal growth factor (EGF) (Invitrogen; PMG8043), Noggin, and RSPO (ENR) as previously described.^2^ For Notch inhibition, organoids were treated with 10 μM DAPT (Sigma; D5942) for the indicated times. *Villin-CreERT2;Atoh1*^*fl/fl*^ organoids were kind gift from Dr. Henner Farin. For complete gene deletion, *Villin-CreERT2;Atoh1*^*fl/fl*^ organoids were treated with 1 *μ*M 4-hydrotamoxifen (4-OHT) for 24 hours, followed by 48 hours of 10 μM DAPT treatment for Notch inhibition. For the RSPO-low challenge experiments, organoids were plated in ENR containing 5% or 2% RSPO.

### Organoid Colony Formation Assay

Organoids were dissociated using ACCUMAX (Merck, Kenilworth, NJ; SCR006) and counted. A total of 2000 single cells were seeded in BME (Cultrex, Minneapolis, MN) per well in a 48-well plate and placed in a 37°C incubator to polymerize for 30 minutes; 300 μL of small intestinal organoid growth media (see previously) plus Rho kinase inhibitor Y-27632 (Sigma-Aldrich; Y0503) was added and cultured for the indicated times. Number of spheres formed in each well was counted as plating efficiency.

### Human Organoids Culture

Samples have been harvested during surgeries at the Great Ormond Street Hospital, London, in accordance with ethical approval, REC Ref: 11/LO/1522. Written informed consent was obtained. Intestinal samples were obtained from patients with various intestinal diseases. Crypts were isolated from human intestinal tissue by incubating for 1 hour with chelation buffer (5.6 mM Na_2_HPO_4_, 8 mM KH_2_PO_4_, 96 mM NaCl, 1.6 mM KCl, 44 mM sucrose, 54.8 mM D-sorbitol, 0.5 M EDTA, and 1M DTT) at 4°C, and plated in drops of BME. After polymerization, culture media was added. Human intestinal organoid media contains advanced Dulbecco’s modified Eagle’s medium (DMEM)/F12 medium (Invitrogen; 12634010) including B27 (Invitrogen; 17504044), nicotinamide (Sigma-Aldrich; N3376), N-acetylcysteine (Sigma-Aldrich; A7250), EGF (Invitrogen; PMG8043), transforming growth factor-β type I receptor inhibitor A83-01 (Tocris, Bristol, UK; 2939), P38 inhibitor SB202190 (Sigma-Aldrich; S7067), gastrin I (Sigma-Aldrich; G9145), Wnt3a conditioned media (50% produced using stably transfected L cells), Noggin, and RSPO conditioned media.

### Disaccharidase Activity Assay

Organoids were washed twice with PBS and incubated with a 56 mM solution of sucrose for 1 hour. Supernatants were collected and frozen until the assay was performed. To detect glucose content, Amplex Red Glucose/Glucose Oxidase Assay Kit (Invitrogen; A22189) was used. Samples were diluted when necessary and incubated with the reaction buffer containing Amplex Red, horseradish peroxidase, and glucose oxidase. Fluorescence was measured in a microplate reader with an excitation wavelength of 540 nm and fluorescence emission detection at 590 nm. Glucose concentration was assessed using a glucose standard curve from 0 to 200 μM.

### FACS Sorting

Crypts were harvested from the proximal jejunum (∼10 cm) by 30-minute incubation in ice-cold 5 mM EDTA/PBS and filtered through a 70-μm strainer. Crypts were dissociated by incubating with Collagenase/Dispase (Roche, Basel, Switzerland; 11097113001) for 20 minutes at 37^o^C, followed by 20-minute incubation with TrypLE (Gibco, Waltham, MA; 12604013) for 20 minutes at 37^o^C. TrypLE was stopped by adding Advanced DMEM (Gibco; 12491015) containing 10% fetal bovine serum (FBS) (Gibco; 10270106) and dissociated cells were passed through a 20-μm strainer. Cells were stained with 4′,6-diamidino-2-phenylindole (DAPI) and resuspended in PBS-0.5% BSA-2 mM EDTA. Cells were separated and re-collected in Advanced DMEM plus 10% FBS based on GFP intensity. Cell sorting was performed on a BD FACSAria II System.

### Cell Culture

HEK293T and LS174T cells were maintained in DMEM GlutaMAX (Gibco; 10566-01) supplemented with 5% FBS (Gibco; 10270106) and 100 units/mL penicillin (Gibco; 15140122) and 100 μg/mL streptomycin (Gibco). Cells were incubated in a humidified atmosphere of 5% CO_2_ at 37°C. For transient overexpression, plasmids were transfected with polyethylenimine (PEI; Polysciences, Warrington, PA; 23966) according to manufacturer’s instructions. For immunoprecipitation (IP) experiments, cells were seeded 24 hours before transfection in a 10-cm plate, using 2 plates per condition and 8 μg of indicated plasmids. For qRT-PCR experiments, cells were seeded 24 hours before transfection in 6-well plates using 1 μg of indicated plasmids.

### Quantitative RT-PCR

RNA was extracted according to the manufacturer’s instructions (Qiagen RNAeasy). Complementary DNA (cDNA) was prepared using High-Capacity cDNA Reverse Transcription Kit (Applied Biosystems, Foster City, CA; #4368813). Quantitative PCR detection was performed using PowerUp SYBR Green Master Mix (Applied Biosystems; A25742). Assays for each sample were run in triplicate and were normalized to housekeeping genes *Ppib* or *β-actin*, where data was expressed as mean ± SEM. Primer sequences are listed in Supplementary Table 6.

### Immunoprecipitation and Immunoblotting

HEK293T cells were treated with doxycycline (Sigma-Aldrich; D9891) 1 μg/mL to induce Flag-MTGs expression for 7 hours before lysate collection. Cells were washed and collected with cold PBS and lysed in cold lysis buffer containing 150 mM NaCl, 30 mM Tris (pH7.5), 1 mM EDTA, 1% Triton X-100, 10% Glycerol, 0.5 mM DTT, protease and phosphatase inhibitor cocktail (Thermo Scientific; 78446). After clarification by centrifugation (18800 g for 30 minutes at 4°C), the cellular lysates were precleared with IgG-agarose beads (Millipore, Bedford, MA; 16-266 for 1 hour at 4°C). Immunoprecipitation of Flag complexes was performed by incubating the cellular lysates with anti-Flag-M2 affinity beads (Sigma; A2220) at 4°C overnight. Immunocomplexes were washed with cold lysis buffer 5 times, resuspended in lysis buffer containing sodium dodecyl sulfate (SDS) sample buffer, and subjected to SDS-polyacrylamide gel electrophoresis and western blot analysis.

### Immunohistochemistry

For analysis of small intestine by immunohistochemistry, tissues were fixed in 10% formalin and embedded in paraffin. Sections were deparaffinized with xylene and rehydrated in a graded series of ethanol. Antigen-retrieval was performed for 20 minutes at high temperature in citrate or tris-EDTA buffer. Slides were then blocked and incubated overnight with anti-Chromogranin A antibody (ab15160, Abcam, Cambridge, UK), anti-lysozyme (A0099; Dako, Glostrup, Denmark), anti-DCAMKL1 (ab37994; Abcam, Cambridge, UK), anti-VILLIN (sc-58897; Santa Cruz Biotechnology, Dallas, TX), anti-MUC2 (sc-15334; Santa Cruz Biotechnology), anti-FABP1 (328607, Thermo Fisher), anti-APOA4 (AF8125, R&D, Minneapolis, MN), anti-RBPJ (RBPSUH [D10A4], Cell Signaling, Danvers, MA; 5313T), anti-GFP (ab6673, Abcam) or negative control at 4°C. Finally, slides were incubated with the secondary antibody for 1 hour, washed 3 times with PBS, incubated with peroxidase substrate, and mounted. For immunofluorescence, slides were incubated with Alexa 488 and Cy5 secondary antibody for 1 hour, washed 3 times with PBS, incubated with DAPI for 15 minutes to visualize nuclear DNA, and mounted with ProLong Gold Antifade Mountant (ThermoFisher; P36934). When indicated, sections were stained for hematoxylin-eosin, alkaline phosphatase and Alcian Blue–Periodic Acid–Schiff staining. EdU was detected according to the manufacturer’s protocol (Click-iT Plus EdU Alexa Fluor 555 imaging kit C 10638, Thermo Fisher Scientific) to evaluate proliferating cell number. Edu+ cells were quantified in at least 10 crypts per mouse (n = 3 mice per group per condition).

### RNAscope

Single-molecule in situ hybridization was performed on mouse intestine as recommended by the manufacturer (ACD; https://acdbio.com, user manual doc. 322310-QKG or 322500 for duplex detection). The probes used were against *MTG8/Runx1t1* (REF 434601), *MTG16/ Cbfa2t3* (REF 474921), *Mtgr1/Cbfa2t2* (REF 491601), *Atoh1* (REF 408791_C2), *Lgr5* (312171), *Olfm4* (REF 311831). Briefly, guts were fixed in formalin O/N, paraffin-embedded and cut into 4-μm-thick slices. Target retrieval was performed for 15 minutes, followed by RNAScope Protease Plus incubation for 24 minutes on the formalin-fixed paraffin-embedded Sample Preparation. The counterstaining and mounting of the slides was performed on a Tissue-Tek Prisma staining machine. RNAscope staining quantifications were performed using ZEN blue software in images acquired at ×40 with a Zeiss Axio Scan.Z1 Slide Scanner. RNAscope score of 1 was assigned to the cells with 1 to 3 dots of staining. Score 2 was for cells with bigger number of dots and/or cluster of dots bigger than 3 μm^2^.

For combined RNAscope and immunofluorescence of *Mtg16* and Muc2, samples were first stained for *Mtg16* using the red channel of duplex RNAscope kit, followed by anti-Muc2 immunostaining as described previously.

### ChIP-seq

Isolated mouse crypts were dual cross-linked first in 2 mM Di(N-succinimidyl) glutarate (DSG; Sigma-Aldrich; 80424) for 45 minutes at room temperature followed by incubation in 1% formaldehyde for 10 minutes as previous described.^3^ The fixation was terminated by quenching with glycine (Sigma-Aldrich; 50046) for 5 minutes at room temperature. The samples were washed twice in PBS and resuspended in commercial lysis buffer containing protease inhibitors from the MAGnify Chromatin Immunoprecipitation System kit (Thermo Scientific; 492024). The chromatin was sheared using the Covaris S2 sonicator (Covaris, Woburn, MA) at 4°C for 10 minutes with the following settings; duty cycle 5%, intensity: 2, cycles per burst: 200, cycle time: 60 seconds. The sonicated chromatin was incubated for one hour at 4°C with Dynabeads protein A/G beads from the kit coupled to 10 μL of anti-MTG16 antibody (Abcam; ab33072) per IP; 10% of the chromatin was used for the Inputs control. The beads were washed with buffers supplied with the kit and samples were de-crosslinked in buffer containing Proteinase K at 55°C for 15 minutes according to the manufacturer’s instructions. The DNA was purified using the DNA Purification Magnetic Beads supplied with the kit and the eluted DNA was verified using the Agilent Bioanalyzer. The DNA library for sequencing was prepared using 20 ng of ChIP’ed DNA for the Kapa Hyper Prep kit with 16 cycles of PCR amplification. The quality of the final DNA library was confirmed on the Agilent Tapestation before the samples were submitted to sequencing on the HiSeq 4000 (Illumina, San Diego, CA).

### ChIP-seq Analysis

ChIP sequencing typically generated ∼27 million 101 base pair paired-end reads per sample. Adapter trimming was performed with cutadapt (version 1.9.1)^4^ and the resulting reads were mapped to the mouse mm10 genome using BWA (version 0.6.2).^5^ Reads that were properly paired, uniquely mapped, and had an insert size ≤2 kb were kept for further analysis.

Genome-wide peak calling was performed with MACS2 callpeak (version 2.1.1.20160309).^6^ Peaks common to both Mtg16 replicates were carried forward for further analysis. Motif discovery was carried out with the HOMER findMotifsGenome.pl program (version 4.8)^7^ using its in-built mm10 annotation with a peak size of ±50 base pairs around the peak summit. The ChIP-Enrich package (version 1.10.0)^8^ was used for gene set enrichment analysis relative to Gene Ontology (GO) biological process. Heatmaps and metaprofiles were generated with deepTools (version 2.5.3).^9^ A scatterplot was generated to show the correlation between the union set of peaks from both Mtg16 ChIP-seq replicates. Read counting was performed with featureCounts,^10^ and normalization was performed with the normalize.quantiles function in R.

Atoh1 and DNase I hypersensitive data were obtained from the Gene Expression Omnibus database (GSE51464),^11^ and reanalyzed in line with the methods described previously.

### RNA-seq and Bioinformatics Analysis

RNA was extracted from isolated mouse crypts of full-length small intestine of 3 WT and 3 *Mtg16 −/−* mice according to the manufacturer’s instructions (Qiagen RNAeasy). RNA integrity was examined using Bioanalyzer 2100 RNA 6000 Nano kit from Agilent. Libraries were generated according to manufacturer’s instructions (KAPA RNA HyperPrep with RiboErase (HMR) - KK8561) and 200 ng RNA input.

RNA sequencing was carried out on the Illumina HiSeq 2500 platform and typically generated ∼20 million 76 base pair strand-specific single-end reads per sample. Adapter trimming was performed with cutadapt (version 1.9.1)^4^ with parameters “--minimum-length=25 --quality-cutoff=20 -a AGATCGGAAGAGC”. The RSEM package (version 1.3.0)^12^ in conjunction with the STAR alignment algorithm (version 2.5.2a)^13^ was used for the mapping and subsequent gene-level counting of the sequenced reads with respect to mm10 RefSeq genes downloaded from the UCSC Table Browser^14^ on December 11, 2017. The parameters used were “--star-output-genome-bam --forward-prob 0”. Differential expression analysis was performed with the DESeq2 package (version 1.12.3)^15^ within the R programming environment (version 3.3.1). An adjusted *P* value (FDR, false discovery rate) of ≤0.1 was used as the significance threshold for the identification of differentially expressed genes.

Gene set enrichment analysis (GSEA) (version 2.2.3)^16^ pre-ranked analysis was performed using the Wald statistic with respect to MSigDB (version 6.1) C2 canonical pathways, C5 GO biological process, and custom signatures obtained from the literature. All parameters were kept as default except for enrichment statistic (classic), min size (5), and max size (50,000).

**Author names in bold designate shared co-first authorship.**

## References

[1] Bjerknes M., Cheng H. (1981). The stem-cell zone of the small intestinal epithelium. V. Evidence for controls over orientation of boundaries between the stem-cell zone, proliferative zone, and the maturation zone. Am J Anat.

[2] Tetteh P.W., Farin H.F., Clevers H. (2015). Plasticity within stem cell hierarchies in mammalian epithelia. Trends Cell Biol.

[3] Yang Q., Bermingham N.A., Finegold M.J. (2001). Requirement of Math1 for secretory cell lineage commitment in the mouse intestine. Science.

[4] van Es J.H., van Gijn M.E., Riccio O. (2005). Notch/gamma-secretase inhibition turns proliferative cells in intestinal crypts and adenomas into goblet cells. Nature.

[5] VanDussen K.L., Samuelson L.C. (2010). Mouse atonal homolog 1 directs intestinal progenitors to secretory cell rather than absorptive cell fate. Dev Biol.

[6] Noah T.K., Donahue B., Shroyer N.F. (2011). Intestinal development and differentiation. Exp Cell Res.

[7] Stamataki D., Holder M., Hodgetts C. (2011). Delta1 expression, cell cycle exit, and commitment to a specific secretory fate coincide within a few hours in the mouse intestinal stem cell system. PLoS One.

[8] Kim T.H., Li F., Ferreiro-Neira I. (2014). Broadly permissive intestinal chromatin underlies lateral inhibition and cell plasticity. Nature.

[9] Philpott A., Winton D.J. (2014). Lineage selection and plasticity in the intestinal crypt. Curr Opin Cell Biol.

[10] Artavanis-Tsakonas S., Rand M.D., Lake R.J. (1999). Notch signaling: cell fate control and signal integration in development. Science.

[11] van Es J.H., Sato T., van de Wetering M. (2012). Dll1+ secretory progenitor cells revert to stem cells upon crypt damage. Nat Cell Biol.

[12] Jadhav U., Nalapareddy K., Saxena M. (2016). Acquired tissue-specific promoter bivalency is a basis for PRC2 necessity in adult cells. Cell.

[13] Jadhav U., Saxena M., O’Neill N.K. (2017). Dynamic reorganization of chromatin accessibility signatures during dedifferentiation of secretory precursors into Lgr5+ intestinal stem cells. Cell Stem Cell.

[14] Rossetti S., Hoogeveen A.T., Sacchi N. (2004). The MTG proteins: chromatin repression players with a passion for networking. Genomics.

[15] Barker N., van Es J.H., Kuipers J. (2007). Identification of stem cells in small intestine and colon by marker gene Lgr5. Nature.

[16] Han H., Tanigaki K., Yamamoto N. (2002). Inducible gene knockout of transcription factor recombination signal binding protein-J reveals its essential role in T versus B lineage decision. Int Immunol.

[17] el Marjou F., Janssen K.P., Chang B.H. (2004). Tissue-specific and inducible Cre-mediated recombination in the gut epithelium. Genesis.

[18] Munoz J., Stange D.E., Schepers A.G. (2012). The Lgr5 intestinal stem cell signature: robust expression of proposed quiescent ‘+4’ cell markers. EMBO J.

[19] Shimizu H., Okamoto R., Ito G. (2014). Distinct expression patterns of Notch ligands, Dll1 and Dll4, in normal and inflamed mice intestine. PeerJ.

[20] Davis J.N., McGhee L., Meyers S. (2003). The ETO (MTG8) gene family. Gene.

[21] Chyla B.J., Moreno-Miralles I., Steapleton M.A. (2008). Deletion of Mtg16, a target of t(16;21), alters hematopoietic progenitor cell proliferation and lineage allocation. Mol Cell Biol.

[22] Fre S., Hannezo E., Sale S. (2011). Notch lineages and activity in intestinal stem cells determined by a new set of knock-in mice. PLoS One.

[23] Calabi F., Pannell R., Pavloska G. (2001). Gene targeting reveals a crucial role for MTG8 in the gut. Mol Cell Biol.

[24] Yu S., Tong K., Zhao Y. (2018). Paneth cell multipotency induced by Notch activation following injury. Cell Stem Cell.

[25] Amann J.M., Chyla B.J., Ellis T.C. (2005). Mtgr1 is a transcriptional corepressor that is required for maintenance of the secretory cell lineage in the small intestine. Mol Cell Biol.

[26] Poindexter S.V., Reddy V.K., Mittal M.K. (2015). Transcriptional corepressor MTG16 regulates small intestinal crypt proliferation and crypt regeneration after radiation-induced injury. Am J Physiol Gastrointest Liver Physiol.

[27] Sansom O.J., Reed K.R., Hayes A.J. (2004). Loss of Apc in vivo immediately perturbs Wnt signaling, differentiation, and migration. Genes Dev.

[28] Haber A.L., Biton M., Rogel N. (2017). A single-cell survey of the small intestinal epithelium. Nature.

[29] Lo Y.H., Chung E., Li Z. (2017). Transcriptional regulation by ATOH1 and its target SPDEF in the intestine. Cell Mol Gastroenterol Hepatol.

[30] Qi Z., Li Y., Zhao B. (2017). BMP restricts stemness of intestinal Lgr5(+) stem cells by directly suppressing their signature genes. Nat Commun.

[31] Schuijers J., Junker J.P., Mokry M. (2015). Ascl2 acts as an R-spondin/Wnt-responsive switch to control stemness in intestinal crypts. Cell Stem Cell.

[32] Moore A.C., Amann J.M., Williams C.S. (2008). Myeloid translocation gene family members associate with T-cell factors (TCFs) and influence TCF-dependent transcription. Mol Cell Biol.

[33] Bjerknes M., Cheng H. (1981). The stem-cell zone of the small intestinal epithelium. I. Evidence from Paneth cells in the adult mouse. Am J Anat.

[34] Sato T., van Es J.H., Snippert H.J. (2011). Paneth cells constitute the niche for Lgr5 stem cells in intestinal crypts. Nature.

[35] Fukuda M., Mizutani T., Mochizuki W. (2014). Small intestinal stem cell identity is maintained with functional Paneth cells in heterotopically grafted epithelium onto the colon. Genes Dev.

[36] Kim T.H., Escudero S., Shivdasani R.A. (2012). Intact function of Lgr5 receptor-expressing intestinal stem cells in the absence of Paneth cells. Proc Natl Acad Sci U S A.

[37] Durand A., Donahue B., Peignon G. (2012). Functional intestinal stem cells after Paneth cell ablation induced by the loss of transcription factor Math1 (Atoh1). Proc Natl Acad Sci U S A.

[38] Kim T.H., Saadatpour A., Guo G. (2016). Single-cell transcript profiles reveal multilineage priming in early progenitors derived from Lgr5(+) intestinal stem cells. Cell Rep.

[39] de Lichtenberg KH, Seymour PA, Jørgensen MC, et al. Notch controls multiple pancreatic cell fate regulators through direct Hes1-mediated repression. 2018. Available at: 10.1101/336305. Accessed September 4, 2020.

[40] Doyen CM, Depierre D, Yatim A, et al. NOTCH assembles a transcriptional repressive complex containing NuRD and PRC1 to repress genes involved in cell proliferation and differentiation. 2019. Available at: 10.1101/513549. Accessed September 4, 2020.

[41] Ladi E., Nichols J.T., Ge W. (2005). The divergent DSL ligand Dll3 does not activate Notch signaling but cell autonomously attenuates signaling induced by other DSL ligands. J Cell Biol.

[42] Williams C.S., Bradley A.M., Chaturvedi R. (2013). MTG16 contributes to colonic epithelial integrity in experimental colitis. Gut.

[43] McDonough E.M., Barrett C.W., Parang B. (2017). MTG16 is a tumor suppressor in colitis-associated carcinoma. JCI Insight.

[44] Parang B., Bradley A.M., Mittal M.K. (2016). Myeloid translocation genes differentially regulate colorectal cancer programs. Oncogene.

