# Supplementary material for: The Transcription Co-Repressors MTG8 and MTG16 Regulate Exit of Intestinal Stem Cells From Their Niche and Differentiation Into Enterocyte vs Secretory Lineages
Source: Gastroenterology. 2020 Oct;159(4):1328–1341.e3. doi: 10.1053/j.gastro.2020.06.012 (PMC7607384; doi:10.1053/j.gastro.2020.06.012)
Supplement: Supplementary Data [file mmc1.pdf]

Supplementary Figure 1

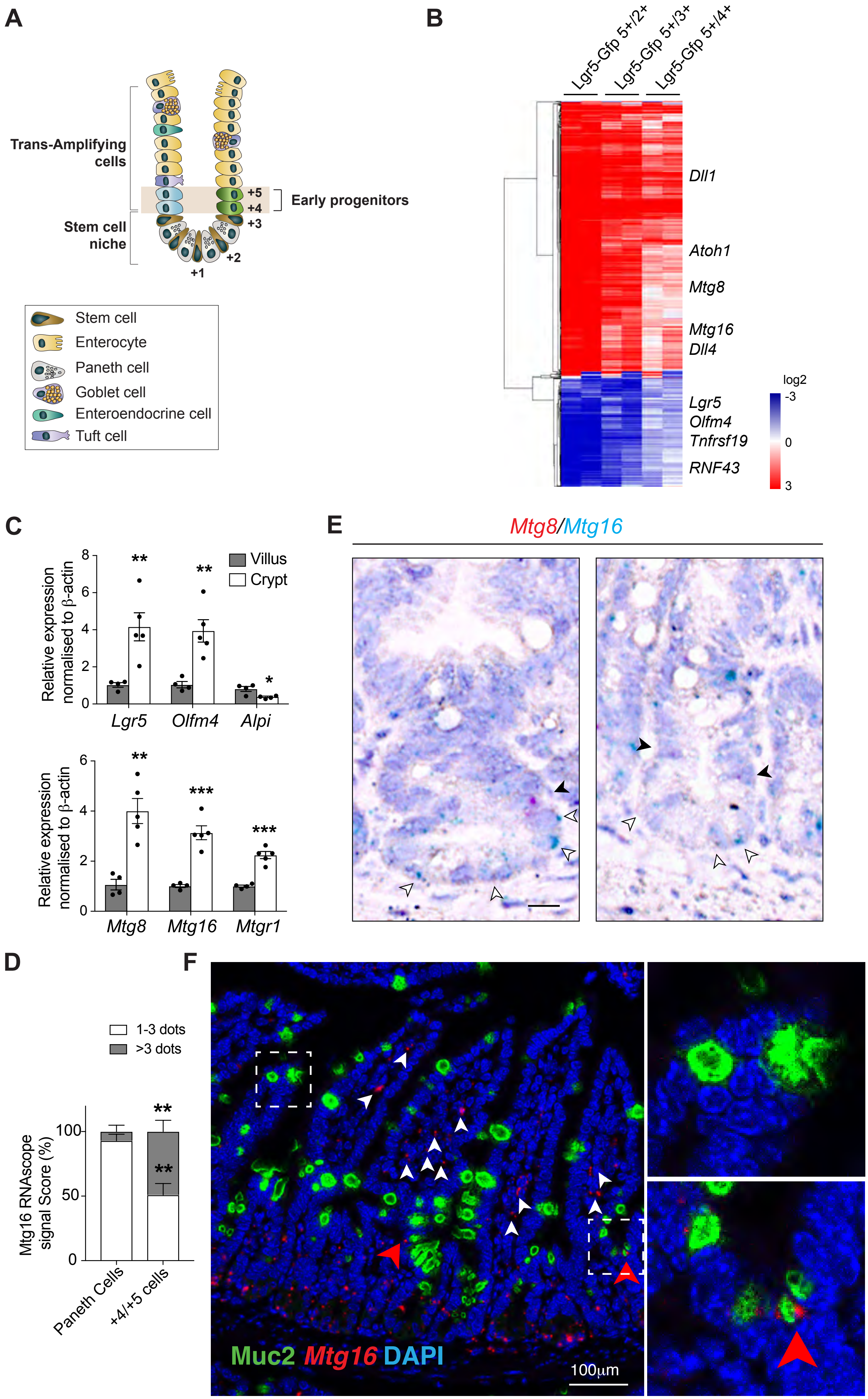

Supplementary Figure 2

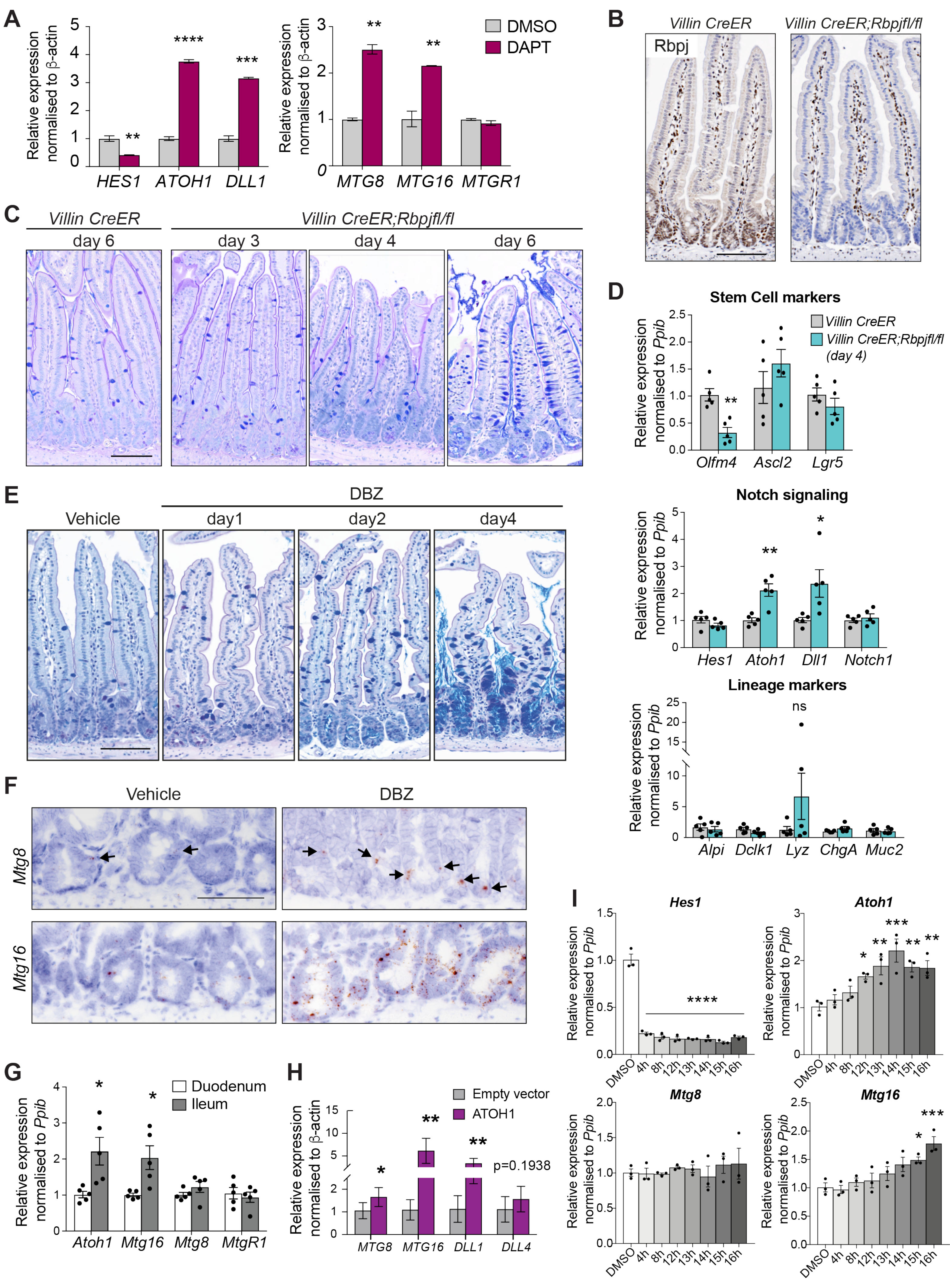

Supplementary Figure 3

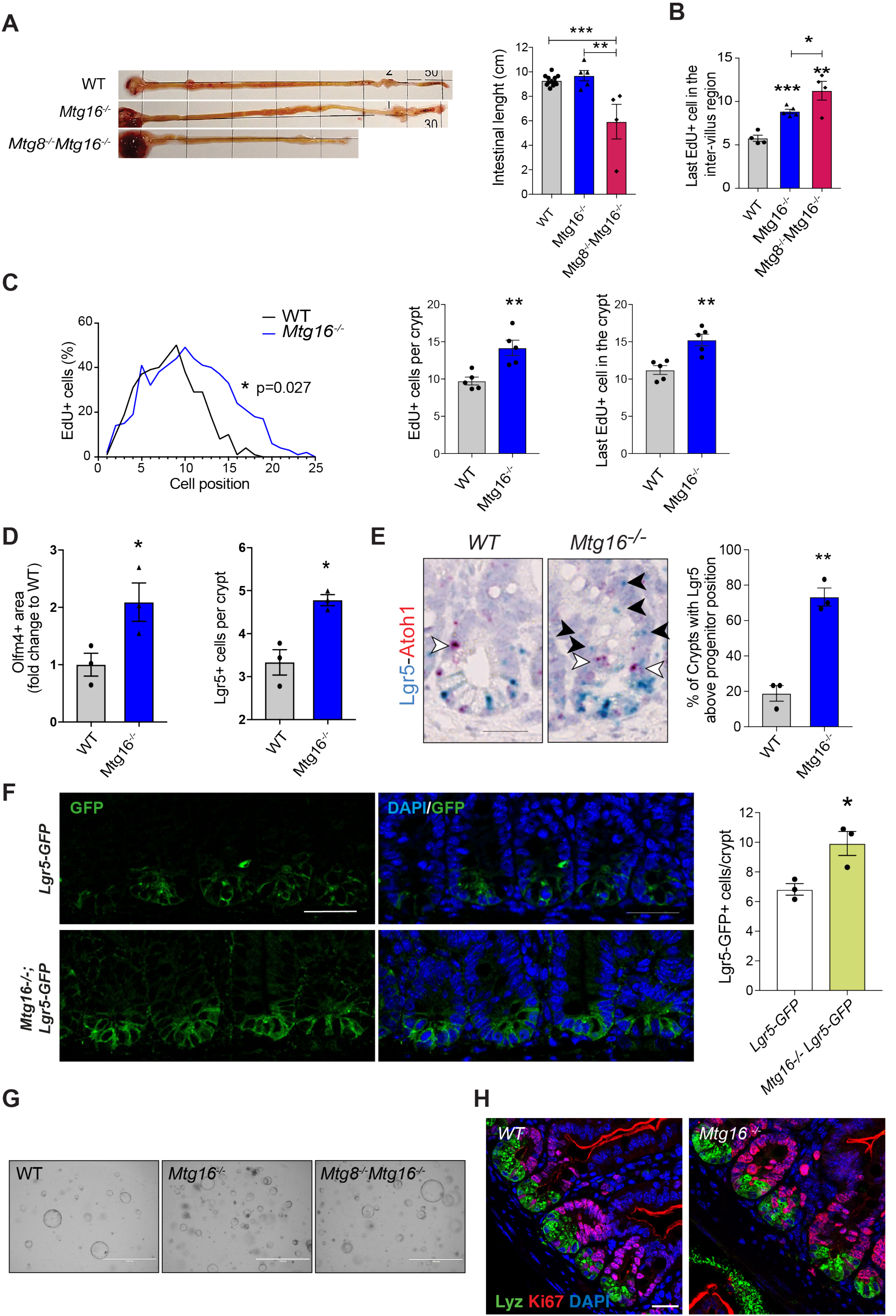

Supplementary Figure 4

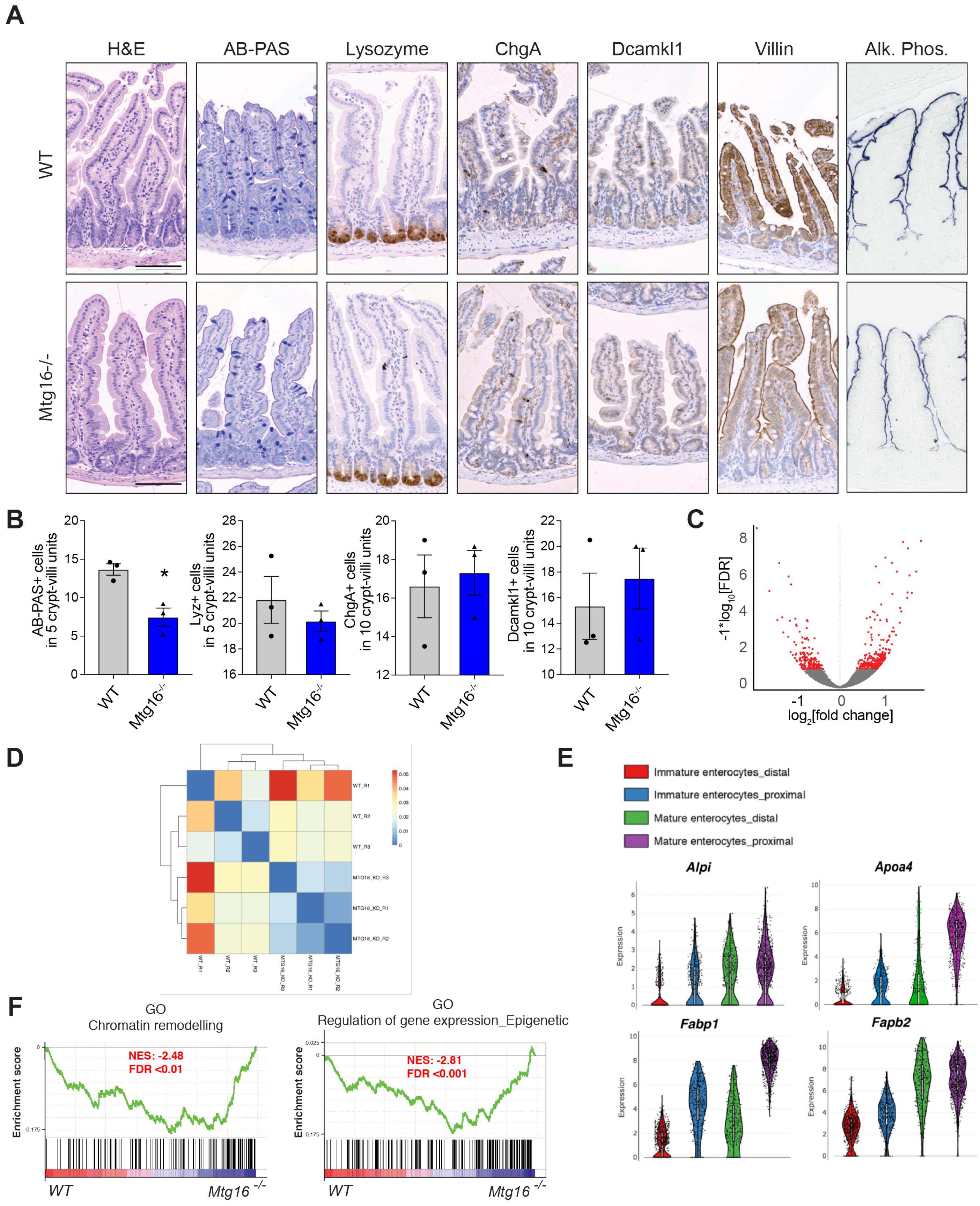

Supplementary Figure 5

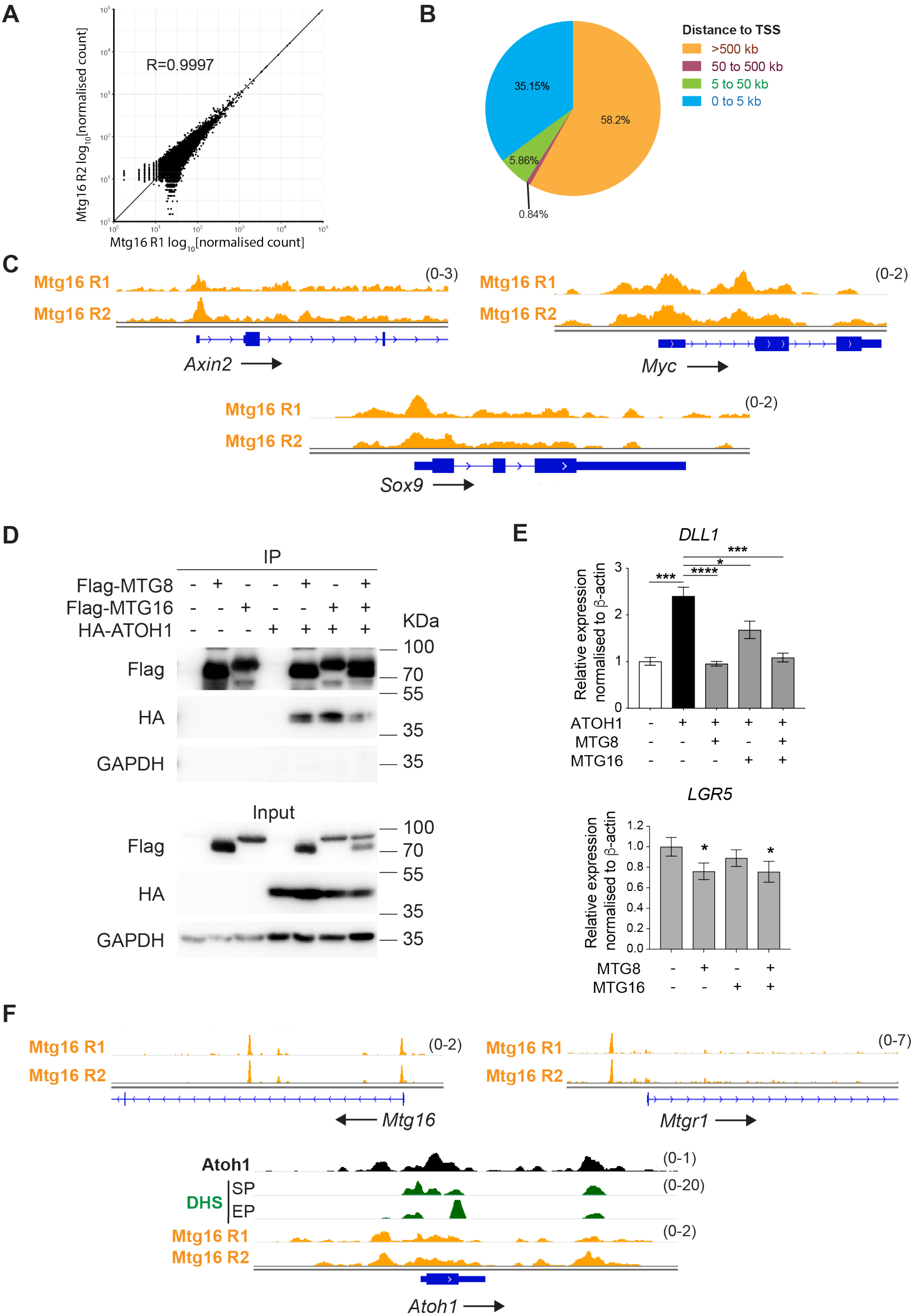

Supplementary Figure Legends

Supplementary Figure 1. *Mtg8* and *Mtg16* are co-expressed at the +4/5 cells upon niche exit. (A) Intestinal crypt structure with defined ISC compartment and trans-amplifying (TA) region. ISCs, located at the stem cell zone intermingled between Paneth cells, divide and give rise to early progenitors at the +4/5 cell positions. These early progenitor cells will differentiate into either absorptive enterocytes or secretory goblet, enteroendocrine, tuft, or Paneth cells. (B) Heatmap showing hierarchical clustering of 525 genes that were enriched in sorted LGR5-GFP-low populations. (C) qRT-PCR analysis of indicated genes in isolated crypts and villi from WT mice. Data are mean ± s.e.m. from biologically independent animals (n=4). *P < 0.05, **P < 0.01, ***P < 0.001, two-sided t-test. (D) Quantitation of *Mtg16* RNAscope signal (by number of dots) in Paneth cells and +4/+5 progenitor cells shown in Figure1B. Data are mean ± s.e.m. from biologically independent animals (n=3). **P < 0.01, two-sided t-test. (E) RNAscope duplex staining of *Mtg8* (in red) and *Mtg16* (in blue) in WT intestinal crypt. The dark arrows indicate co-localized staining. Scale bar, 20μm. (F) Co-immunofluorescence staining of MUC2 (green) and *Mtg16* (red, RNAscope). Red arrows indicate co-staining and white arrows indicate *Mtg16* stromal staining. Scale bar, 100μm.

Supplementary Figure 2. *Mtg8* and *Mtg16* are repressed by Notch. (A) Human intestinal organoids were treated with DAPT for 4 days and mRNA level of indicated genes were measured by qRT-PCR. Data represent mean ± s.e.m. of two independent experiments. *P < 0.05, **P < 0.01, ***P < 0.001, two-sided t-test. (B) RBPJ staining in intestinal tissue from *Villin CreER* and *Villin CreER;Rbpjfl/fl* mice collected at day4 after tamoxifen induction. Scale bar, 100μm. (C) AB-PAS staining of small intestinal tissue from *Villin CreER* and *Villin CreER;Rbpjfl/fl* mice collected at day 3, 4 or 6 after tamoxifen induction. Scale bar, 100μm. (D) qRT-PCR analysis of stem cell markers, notch signaling pathway and lineage markers of intestinal epithelium from *Villin CreER* and *Villin CreER;Rbpjfl/fl* mice collected at day4 after tamoxifen induction. Data represent mean ± s.e.m. from biologically independent animals (n=4 per group). Three independent experiments were performed. *P < 0.05, **P < 0.01, ***P < 0.001, two-way ANOVA. (E) AB-PAS staining of small intestinal tissue from WT mice collected at day 1, 2 or 4 after DBZ or vehicle treatment. Scale bar, 100μm. (F) RNAscope brown staining of *Mtg8* and

Mtg16 in intestinal tissue obtained from WT mice collected 2 days after DBZ or vehicle treatment. Arrows indicate *Mtg8*+ cells. Scale bar, 50µm. (G) qRT-PCR analysis of the indicated genes in proximal and distal parts of adult mouse intestine. Data represent mean ± s.e.m. from biological replicates (n=5). *P < 0.05, two-sided t-test. (H) qRT-PCR analysis of the indicated genes upon ATOH1 overexpression in HEK293T cells. Three independent experiments were performed *P < 0.05, **P < 0.01, ***P < 0.001, two-sided t-test. (I) qRT-PCR analysis of WT mouse intestinal organoids treated with Notch inhibitor DAPT at different time points. Data represent mean ± s.e.m. from biological replicates (n=3). The experiment was performed twice. *P < 0.05, **P < 0.01, ***P < 0.001, two-sided t-test.

Supplementary Figure 3. MTG8 and MTG16 repress crypt proliferation and ISC genes. (A) Intestinal length measurements of WT, *Mtg16-/-* and *Mtg8-/-Mtg16-/-* P0 pups showing significant shorter intestine upon *Mtg8* loss. Data represent mean ± s.e.m. of three independent experiments (n=4 to 12 mice per group). *P < 0.05, **P < 0.01, ***P < 0.001, 1-way ANOVA. (B) Quantitation of the last EdU+ cell in the inter-villus region of Figure3A. *P < 0.05, **P < 0.01, ***P < 0.001, 1-way ANOVA. (C) Quantitation of Edu staining in Figure 3C. Graphs showing position of the last EdU+ cells distribution along the crypt, quantitation of EdU+ cells per crypt and the position of the last EdU+ cell in the crypt. Data represent mean ± s.e.m. (n=5 mice per group). At least 10 representative crypts per animal have been analyzed. *P < 0.05, **P < 0.01, ***P < 0.001, two-sided t-test. (D) Quantitation of RNAscope staining in Figure3D. (E) Representative images showing *Lgr5* (blue) and *Atoh1* (red) duplex RNAscope staining in WT and *Mtg16-/-* adult intestinal crypts. The graph shows percentage of crypts with *Lgr5* staining above (black arrows) the *Atoh1*+ progenitor cells (white arrows). Data represent mean ± s.e.m. (n=3 mice per group). 30 crypts analyzed per animal. *P < 0.05, **P < 0.01, ***P < 0.001, two-sided t-test. Scale bar, 20µm. (F) GFP staining (green) in intestinal tissue from *Lgr5-GFP* and *Mtg16-/-*;*Lgr5GFP* mice and its quantification of LGR5-GFP+ cells per crypt. Data represent mean ± s.e.m. (n=3 mice per group). 15 crypts analyzed per animal. *P < 0.05, **P < 0.01, ***P < 0.001, two-sided t-test. Scale bar, 50µm. (G) Representative images of WT, *Mtg16-/-* and *Mtg8-/-Mtg16-/-* organoids from the colony formation assay shown in Figure 3F. Scale bar, 1000µm. **(**H**)** Immunofluorescence staining of Lyz (green) and Ki67 (red) in WT and *Mtg16-/-* adult intestinal tissues. Scale bar 50µm.

Supplementary Figure 4. Loss of MTG8/16 impairs intestinal lineage specification. (A) H&E, AB-PAS (Goblet cells), Lyzozyme (Paneth cells), CHGA (enteroendocrine cells), DCAMLK1(Tuft cells), Villin and Alkaline phosphatase (enterocytes) stainings in WT and *Mtg16*-/- adult proximal intestinal tissue. (B) Quantitation of AB-PAS, Lysozyme, CHGA and DCAMKL1 staining shown in (A). At least 10 representative crypts per animal have been analyzed. *P < 0.05, **P < 0.01, ***P < 0.001, two-sided t-test. (C) Volcano plot displaying 478 differentially expressed genes upon *Mtg16* deletion with cut-off q-value (FDR; False Discovery Rate) <0.1 based on RNA-seq analysis of intestinal crypts isolated from adult animals. (D) Heatmap showing three wild type mice and three *Mtg16-/-* mice used for RNA-seq analysis based on clustering of top-500 genes with the highest variability across all samples. (E) Differential expression of various enterocyte markers *Alpi*, *Apoa4*, *Fabp1* and *Fabp2* mature and immature enterocyte populations in the intestine. Data extracted from: https://portals.broadinstitute.org/single_cell/study/SCP44/small-intestinal-epithelium[28]. (F) GSEA showing enrichment of chromatin remodeling and epigenetic regulation of gene expression upon deletion of the transcriptional co-repressor Mtg16.

Supplementary Figure 5. MTG16 transcriptional control of ISC fate and lateral inhibition. (A) Correlation plot of MTG16 ChIP-seq biological replicates. (B) Pie chart showing percentage of the 478 differentially expressed genes upon *Mtg16* deletion that harbors Mtg16-binding sites (ChIP-seq data) with indicated distance to the transcription start site (TSS). (C) ChIP-seq data showing Mtg16 binding signal (per million reads) to the promoter regions of Wnt target genes *Axin2*, *Myc* and *Sox9*. (D) HEK293T cells were transfected with HA-tagged ATOH1, flag-tagged MTG8 or MTG16. Lysates were subjected to anti-Flag IP followed by western blotting using the indicated antibodies. (E) qRT-PCR analysis of *DLL1* and *LGR5* expression in HEK293T cells overexpressed with the indicated plasmids. *P < 0.05, **P < 0.01, ***P < 0.001, 1-way ANOVA for DLL1 and two-sided t-test for LGR5. (F) MTG16 binding signal (per million reads) to its own locus and promoter regions of *Mtgr1* and *Atoh1*.
